# Hidden toxicities: assessing city-wide contamination by potentially toxic elements from illegal dumping in Jackson, Mississippi

**DOI:** 10.1007/s10653-026-03450-y

**Published:** 2026-09-24

**Authors:** Erica D. Walker, Craig Klevan, Kelsey Mathews, Sai Venkat Mandalapu, Cristina Nica, Naira Ibrahim

**Affiliations:** 1 Community Noise Lab, Department of Epidemiology, Brown University School of Public Health, Providence, RI 02903 USA; 2 Geosyntec Consultants, Columbia, MD 21044 USA; 3 Department of Biology, College of Science, Engineering, and Technology, Jackson State University, Jackson, Mississippi USA

**Keywords:** Urban soil contamination, Potentially toxic elements, Contamination factor, Portable X-ray fluorescence, Illegal dumping, Lead exposure

## Abstract

Illegal dumping is widespread where waste collection is unreliable, and municipal responses usually end with removal of visible waste, leaving the underlying soil unexamined. We asked whether elemental enrichment persists below the surface once waste is cleared, whether lithogenic and waste-derived contributions can be separated using field-portable screening alone, and whether a low-cost protocol can prioritize sites in a city without laboratory capacity. At 45 community-reported dumping sites across Jackson, Mississippi, we measured eight potentially toxic elements (the metalloid arsenic with cadmium, chromium, copper, manganese, nickel, lead and zinc) in composite soils at 0 to 2.5 cm and 15 cm by portable X-ray fluorescence, with laboratory ICP-OES on a 13-site subset for independent method comparison. Lead, copper and zinc dominated enrichment and co-occurred, and concentrations at 15 cm approached surface values, indicating the elemental burden is not lifted when surface waste is removed. Principal component and cluster analyses, corroborated by partial-extraction recoveries, separated a chromium, nickel and iron association consistent with lithogenic sources from a zinc, arsenic and lead association consistent with waste inputs. Distributions were strongly right-skewed, so ward means reflect a few extreme sites. A screening-level risk assessment gave hazard indices above unity at 21 of 45 sites for a child resident, driven largely by geogenic arsenic and manganese, whereas lead, the clearest waste-derived element, exceeded the United States Environmental Protection Agency residential soil screening level at 11 sites. Field screening with selective laboratory confirmation offers resource-limited cities a practical route to prioritize remediation.

**Supplementary Information:**

The online version contains supplementary material available at https://doi.org/10.1007/s10653-026-03450-y.

## Introduction

Waste is produced everywhere people live, and the volume keeps rising. The United Nations Environment Programme estimates that the world generates about 2.1 billion tons of municipal solid waste each year, a figure projected to reach 3.8 billion tons by 2050, an increase of roughly 81% (UNEP, 2024). The United States accounts for an outsized share of this total, generating approximately 292.4 million tons annually, or about 14% of the global figure (U.S. Environmental Protection Agency, 2020a). Where collection and disposal systems work, waste is processed and stays largely out of sight. Where those systems falter, the consequences surface quickly, and one of the most immediate is illegal dumping.

Illegal dumping is the informal disposal of waste in vacant lots, abandoned properties, roadsides, and other overlooked spaces. The material left at these sites is rarely limited to household garbage; it routinely includes tires, furniture, construction and demolition debris, yard waste, electronics, and chemical containers (U.S. Environmental Protection Agency, 1998). What makes this more than an aesthetic problem is what happens after the waste is dropped. As mixed materials sit in contact with bare soil and weather in place, they can release metals and other contaminants into the ground, so the environmental footprint of a site often extends well beyond what is visible at the surface (Ihedioha et al., 2017; Vongdala et al., 2019). Contamination by potentially toxic elements is a particular concern at sites that accumulate construction debris, electronic waste, and tires.

Potentially toxic elements are persistent. Unlike organic contaminants they are not degraded by microbial or chemical breakdown, so their residence times in surface soils are very long, although they are not immutable: they bind to soil particles, migrate into deeper horizons, enter runoff and groundwater, or are taken up by plants. Once in the soil, their behavior is governed by local geochemistry, including pH, texture, organic matter, mineralogy and microbial activity, all of which shape mobility, bioavailability and toxicity (Kabata-Pendias, 2011; Tchounwou et al., 2012). Because these elements remain in place and continue to transform chemically, a contaminated soil represents a long-term environmental and public health liability rather than a transient one. Elements mobilized from contaminated soils can also be transferred to plants and enter the food chain, and the resulting health risk depends on the quantity ingested, with children generally more vulnerable than adults (Senila et al., 2024; Şenilă et al., 2012). We follow IUPAC guidance in avoiding the term “heavy metals”, which has no authoritative definition, and use “potentially toxic elements” throughout; arsenic is a metalloid rather than a metal and is included on the basis of its toxicological relevance (Duffus, 2002; Madrid, 2010; Pourret & Bollinger, 2018; Pourret et al., 2021).

The elements commonly enriched at dumping sites can often be traced to the materials present. Treated wood, construction debris, and pesticide residues are associated with arsenic and chromium; tires with zinc and copper; wiring, plumbing, and electronics with copper, lead, and cadmium; batteries and painted or demolition debris with lead; and metal scrap with manganese and nickel (Fazzo et al., 2023; Houessionon et al., 2021; Mayer et al., 2024; McKenzie et al., 2009; National Institute of Environmental Health Sciences, n.d.; U.S. Environmental Protection Agency, n.d.-a). Reading the soil signal alongside the waste inventory therefore offers a way to connect a site's contents to its measurable geochemical signature.

Despite this, the typical response to illegal dumping stops at the surface. Where a change of land use or new construction is proposed, formal environmental site assessment including soil analysis is required in many jurisdictions, and such assessment is mandatory in many countries. Informal dumping sites, however, are usually cleared as a sanitation action rather than as a property transaction, so they do not trigger those requirements. Communities remove the visible waste, and former dump sites are frequently repurposed into community gardens, recreational areas or playgrounds without the soil being tested. These actions improve appearance and return land to use, but they do not address what remains below ground. If contaminated soil is never assessed or remediated, a site can look fully recovered while continuing to present exposure pathways to the people who later use it (Mitchell et al., 2014).

Jackson, Mississippi, the setting for this study, illustrates how quickly this problem can compound. The city has struggled for years to maintain reliable waste collection. Since at least 2021, the City Council has been unable to hold a stable trash-pickup vendor, and the dispute culminated in a 2023 breakdown that halted residential collection across the city and strained sanitation infrastructure (Rozier, 2024). Illegal dumping rose sharply during that period. Residents, local media, and city officials have all drawn attention to the problem, yet it remains widespread today, which makes Jackson both an urgent and an instructive place to examine what dumping leaves in the soil.

This study addresses three questions. First, does elemental enrichment persist below the visible surface, and how do concentrations at depth relate to those at the surface? This determines whether removing surface waste is a sufficient remedy. Second, can lithogenic and waste-derived contributions be distinguished using field-portable screening alone, without speciation or sequential extraction? Third, does a low-cost, transferable screening protocol identify priority sites reliably, and where does it fail? To answer these we (i) quantify concentrations of eight potentially toxic elements at reported illegal dumping sites in Jackson using portable X-ray fluorescence, an accessible and lower-cost method; (ii) summarize element-specific enrichment and cumulative burden using the contamination factor (Cf) and the degree of contamination (Cdeg), interpreted as screening indices rather than as measures of risk; (iii) separate source groups using principal component analysis, hierarchical clustering and the recovery ratio between total and partial-extraction measurements; and (iv) place the concentrations in a health-relevant frame using absolute regulatory screening levels and a screening-level risk assessment following United States Environmental Protection Agency guidance. The eight elements were selected because they are associated with the waste streams recorded at these sites, because they are toxicologically significant in residential soil exposure, and because they are determined reliably by the instrument configuration used. All results reported here are available through our public data repository.

To our knowledge, this is the first city-wide assessment in the United States to systematically measure contamination by potentially toxic elements in soils at community-reported illegal dumping sites.

## Materials and methods

### Study area

This study was conducted in Jackson, Mississippi, USA, a city that has experienced sustained challenges with waste management, infrastructure stability and illegal dumping. Dumping sites are distributed across the city's wards and occur in a range of settings, including residential neighborhoods, abandoned properties, vacant lots, roadsides and areas adjacent to commercial or industrial activity. We aggregated measurements to the ward level so that environmental data would align with the administrative units used for governance and planning. Jackson's wards differ substantially in income, poverty rate and racial composition, and a full socioeconomic characterization drawn from the American Community Survey (U.S. Census Bureau, 2022) is provided in Supplementary Table S1; these variables provide context for the study area and are not analyzed here.

### Illegal dumping repository and site identification

**Fig. 1:**
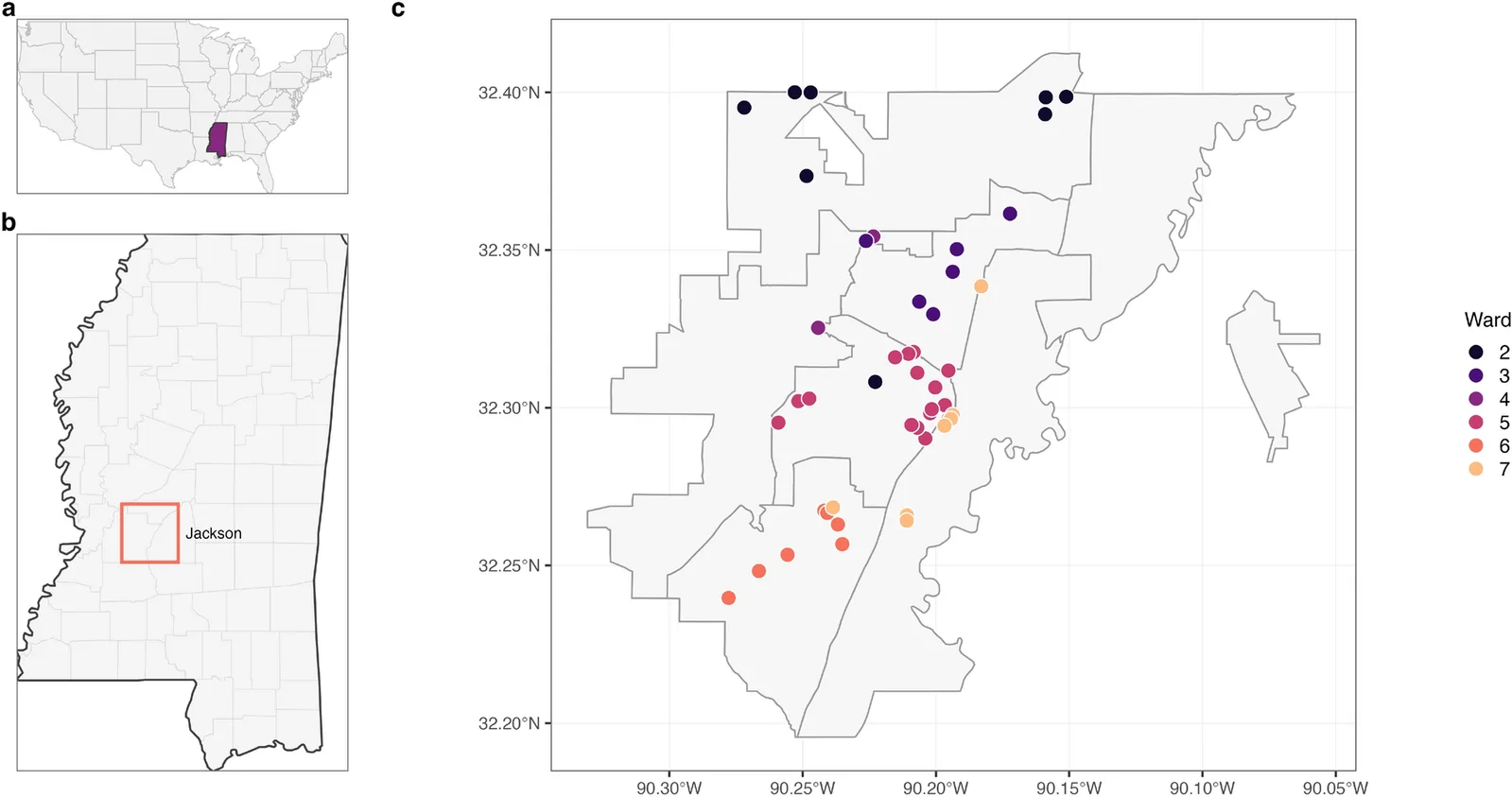
Location of the study area and of cataloged illegal dumping sites. **a** Mississippi within the United States. **b** Jackson within Mississippi. **c** Catalogued illegal dumping sites across the wards of Jackson, Mississippi

Illegal dumping sites were identified through a previously established community-based reporting system and environmental repository. The repository allows residents and field teams to document dumping activity using standardized protocols that capture geographic coordinates, site photographs, waste composition, environmental observations, and exposure-related measurements. It functions as an urban environmental data infrastructure, bringing community observations, field measurements, and spatial visualization together in a single platform accessible to both residents and municipal agencies. Beyond recording where dumping occurs, the system supports environmental characterization and comparison across sites and wards. Sites included in the present study were drawn from the repository on the basis of confirmed dumping activity, accessibility, and suitability for soil sampling. In total, we sampled soil at 45 sites across Jackson (Fig. 1).

### Site characterization

Each site was characterized to record waste composition in one or more of the following categories: tires, domestic waste, construction debris, furniture, yard waste, electronic waste and chemicals (discarded containers and residues of paints, solvents, automotive fluids and similar household or commercial products). These categories were chosen because they capture the dominant waste streams observed at illegal dumping sites and because each is linked to distinct element signatures, allowing visible waste composition to be related to the elements subsequently measured in the soil (Wuana & Okieimen, 2011). Categories were recorded as present or absent rather than quantified by volume or mass. Soil collection took place between 4 June 2024 and 30 June 2025, with 24 of the 45 sites sampled in November 2024. No waste was removed from any site by the study team; sites were sampled as found, with waste present. Consequently the quantity, composition and timing of any subsequent waste removal are not available as study variables. Site-level detail for all 45 sites, including coordinates, sampling date, waste categories, soil properties, element concentrations and indices, is given in Supplementary Tables S4a to S4e.

### Soil sampling

Soil sampling followed U.S. EPA field protocols (U.S. Environmental Protection Agency 2020b). At each site, soil was collected from 20 locations distributed across the dumping area to capture spatial variability. Sampling began at a central reference point, and additional points were established across the site; each location was marked with a field flag to ensure consistent coverage and prevent duplicate collection. At every flagged location, soil was taken at two depths, 0 to 2.5 cm (surface) and 15 cm (subsurface). The two-depth design was intended to test whether contamination was confined to surface deposition or extended deeper, which would point to persistent accumulation from repeated dumping, soil disturbance, and mixing.

After collection, all surface samples from a site were combined in one container to form a composite surface sample, and all 15 cm samples were combined separately to form a composite subsurface sample. Compositing reduces localised heterogeneity and yields a more representative characterization of each site, but it removes within-site variance by construction; subsample volumes were not individually weighed, so the composite represents an approximately equal-volume rather than a strictly equal-mass combination. Samples were transported to the laboratory for preparation and analysis, where soils were manually homogenized and dried in a Model 30 Laboratory Oven (Quincy Lab, Chicago, IL, USA) at 27 °C for 12 h to limit moisture interference. Dried samples were sieved sequentially through a No. 10 (2 mm) sieve to remove coarse material and then through a No. 60 (250 µm) sieve. All analyses, by both portable X-ray fluorescence and inductively coupled plasma optical emission spectrometry, were performed on the resulting sub-250 µm fraction; concentrations were not determined separately in the coarser fraction.

Prepared soil was transferred into XRF sample cups. For each composite sample, three replicate cups were prepared, each holding approximately 5 g of homogenized soil to ensure adequate sample thickness and analytical consistency. Replicate preparation allowed us to assess measurement repeatability and reduce variability from sample heterogeneity. The overall approach was designed to support consistent comparison across heterogeneous dumping sites while remaining accessible, scalable, and practical for broader community and municipal use.

### Potentially toxic element analysis using XRF

Concentrations were measured with a Bruker TRACER 5 g portable X-ray fluorescence (pXRF) spectrometer (Billerica, MA, USA) operated with the manufacturer's factory-installed soil calibration and factory soil method. No user-developed or site-specific empirical calibration was constructed. Portable XRF was chosen because it provides a more accessible and lower-cost route to soil screening than laboratory-intensive methods alone, which makes broader spatial assessment across many distributed sites feasible. Prepared samples were analyzed under controlled laboratory conditions rather than by direct in situ field measurement, which reduces the variability associated with surface readings. Approximately 5 g of prepared soil was placed in each sample cup to ensure adequate sample thickness. Each composite sample was analyzed in triplicate and the replicate mean was used to represent the concentration at that site and depth. Element-specific acquisition times of either 60 or 90 s were used, selected from Bruker recommendations and preliminary trial measurements of signal stability and reproducibility. The elements of concern were arsenic (As), cadmium (Cd), chromium (Cr), copper (Cu), lead (Pb), manganese (Mn), nickel (Ni) and zinc (Zn); total iron (Fe) was also determined and used in the interpretation of element associations.

Study-specific limits of detection and quantification were not independently established, and a complete manufacturer detection-limit table for the TRACER 5 g factory soil calibration was not available. We therefore did not adopt generic application-note or EPA Method 6200 example values as though they were study-specific, and the pXRF results are interpreted as screening-level measurements throughout. Instead, analytical reproducibility was assessed empirically in two ways: by triplicate measurement of every composite, and by comparing the 60 and 90 s acquisitions for every sample. Lead, zinc and manganese reproduced closely between acquisition times (mean relative differences of 3.5 to 7.8%, r > 0.96 in all cases), whereas arsenic, copper and nickel did not (arsenic mean relative difference 21.7% at the surface, with 14 of 45 samples differing by more than 25%). Reproducibility was therefore poorest for the elements present at the lowest concentrations, which is the principal reason the pXRF data are treated as screening-level rather than definitive. The full acquisition-time comparison is given in Supplementary Table S5. No certified reference material was analyzed by pXRF; independent corroboration was obtained instead from the laboratory subset described in Sect. “Laboratory analysis by ICP-OES”.

### Soil properties and setting

Soil pH was recorded at every site using a field probe, and is reported as a field measurement rather than a laboratory determination on a soil-to-water suspension. Total iron was determined by pXRF as described above. Soil series, surface and subsurface texture, organic matter content and carbon stock were obtained by spatially joining each site to the United States Department of Agriculture Natural Resources Conservation Service soil survey; these are mapped survey attributes at map-unit resolution and were not determined on the collected samples. Total organic carbon was not measured on the samples. Field texture descriptions recorded at the time of sampling were used to confirm the survey texture classes. Laboratory granulometry, total organic carbon and leaching or sequential extraction were not performed, so no inference is drawn about element mobility or bioavailability.

### Composite contamination indices

Composite contamination indices were calculated to capture both element-specific enrichment and cumulative element burden at each site. First, the XRF concentrations from the 0 to 2.5 cm and 15 cm depths were combined into a single site-level concentration for each element. For each element i, a depth-integrated mean concentration (C_i,sample_) was calculated with Eq. 1:

1 $$C_{i,sample} { } = { }\frac{{\left( {C_{1,i} { } \times { }2.5{\text{ cm }} + { }\frac{{\left( {C_{1,i} { } + { }C_{2,i} } \right)}}{2}{ } \times { }12.5{\text{ cm}}} \right)}}{{15{\text{ cm}}}}$$

This depth-weighting treats the 0 to 2.5 cm surface value as representative of the uppermost 2.5 cm and the average of the surface and subsurface values as representative of the remaining 12.5 cm of the 0 to 15 cm profile. The resulting concentration for each element was then compared with a reference background value to obtain the contamination factor (Cf). Background values represent expected baseline concentrations for each element in uncontaminated or regional reference soils, and here they were drawn from published soil surveys relevant to Jackson and the surrounding region. Jackson soils are dominated by Peoria Loess, a deep, nearly level, poorly drained, slowly permeable series common in the Southern Mississippi Valley and characterized by high silt content (Soil Survey Staff, 2003). The reference values used for each element are listed in Table 1.

**Table 1:** Reference background values for the potentially toxic elements of concern

Element	Reference value (mg/kg)	References
Cr	37.5	Smith et al. (2017)
Mn	509.0	Pettry and Switzer (1983)
Ni	30.6	Pettry and Switzer (1983)
Cu	14.3	Pettry and Switzer (1983)
Zn	38.9	Pettry and Switzer (1983)
As	6.9	Pettry and Switzer (2001)
Pb	26.3	Pettry and Switzer (1983)

The contamination factor for each element was calculated with Eq. 2:

2 $$C_{{\mathrm{f,i}}} { } = { }\frac{{C_{{\mathrm{i,sample}}} }}{{C_{{\mathrm{i,reference}}} }}$$

where C_f,i_ is the contamination factor for element i, C_i,sample_ is the depth-integrated XRF concentration of element i, and C_i,reference_ is its background value. A Cf above 1 indicates that the measured concentration exceeded background, and larger values indicate greater enrichment. Cf values were grouped into the pollution classes shown in Table 2, following previous studies of soil contamination (Ebong et al., 2019; Ihedioha et al., 2017).

**Table 2:** Contamination factor (Cf) classification

Cf	Significance	Remarks
≤ 1	No contamination (at or below background)	No negative effects on soil, plants, environment
1–2	Slight pollution	May pose negative effects on soil, plants, environment
2–4	Moderate pollution	May pose negative effects on soil, plants, environment
4–8	Severe pollution	Will pose negative effects on soil, plants, environment
> 8	Very severe pollution	Will pose negative effects on soil, plants, environment

The class boundaries and the accompanying remarks are those of the cited sources. In this study the classes are used to rank and compare sites, and not as statements about ecological or health effects, for the reasons given in Sect. “Composite contamination indices”

For each site, the degree of contamination (Cdeg) was then calculated as the sum of the Cf values across all elements of concern, using Eq. 3:

3 $$C_{{{\mathrm{deg}}}} { } = { }\mathop \sum \limits_{i = 1}^{n} C_{f,i}$$

where n is the number of elements summed. Cadmium was determined but was above the pXRF reporting threshold at only one of 45 sites at the surface and three of 45 at 15 cm. Because assigning non-detects a value of zero would allow cadmium to contribute a term at every site while being measured at almost none, cadmium is excluded from Cf and Cdeg and reported descriptively instead. Sect. “Comparison of pXRF with laboratory ICP-OES” shows further that the four pXRF cadmium readings are not corroborated by laboratory analysis, which strengthens the case for excluding the element. The indices therefore use n = 7, corresponding to Cr, Mn, Ni, Cu, Zn, As and Pb, all of which were detected at every site at both depths, so no substitution convention is applied anywhere in the index. An eight-element calculation including cadmium is reported as a sensitivity analysis in Supplementary Table S3.

These indices are used for screening and prioritization only, and not as measures of risk. Enrichment factors and related background-referenced indices have well-documented intrinsic weaknesses: they depend entirely on the chosen reference value, they conflate analytical and geological variability, and the resulting class boundaries are arbitrary (Reimann & de Caritat, 2000). The reference values used here derive from regional pedological surveys of differing vintage, and the analytical basis of the earlier determinations is not fully documented in the original publications. A locally measured population of uncontaminated soils analyzed through cumulative distribution functions would be the stronger design, and we did not collect one. For these reasons all health-related interpretation in this paper rests on absolute regulatory screening levels and on the risk assessment described in Sect. “Statistical analysis and risk assessment”, neither of which requires a background value, and the indices are retained only to rank and compare sites (Table 3).

**Table 3:** Degree of contamination (Cdeg) classification

Cdeg	Degree of contamination
Cdeg < 8	Low degree of contamination
8 ≤ Cdeg < 16	Moderate degree of contamination
16 ≤ Cdeg < 32	Considerable degree of contamination
Cdeg ≥ 32	High degree of contamination

### Laboratory analysis by ICP-OES

To provide an independent laboratory comparison, a subset of soil samples (n = 13, 28.9%) was analyzed by inductively coupled plasma optical emission spectrometry (ICP-OES). Analyses were performed by Waters Agricultural Laboratories, Inc. (Vicksburg, Mississippi, USA) on a PerkinElmer Optima 8000 spectrometer following acid digestion by U.S. EPA Method 3050B (U.S. Environmental Protection Agency, 1996a). Approximately 1 g of dried, homogenized soil was digested per tube, with three replicates per sample. Digestion used 10 mL of 1:1 nitric acid with refluxing at 95 ± 5 °C without boiling, followed by 5 mL of concentrated nitric acid, with further 5 mL additions until brown fumes were no longer produced. After cooling, 2 mL of reagent water and 3 mL of 30% hydrogen peroxide were added, with further peroxide in 1 mL aliquots to a maximum of 10 mL. For the ICP-OES branch of the method, 10 mL of concentrated hydrochloric acid was then added and the digest refluxed for 15 min. Digests were filtered through a 0.45 µm filter, centrifuged to remove residual particulates, and brought to a final volume of 100 mL with reagent water. Instrument operating conditions and the emission wavelength monitored for each element are given in Table 4.

**Table 4:** Operating conditions and emission wavelengths for ICP-OES analysis

Parameter	Value
Instrument	PerkinElmer Optima 8000 ICP-OES
RF power	1300 W
Plasma gas flow	15 L/min argon
Auxiliary gas flow	0.2 L/min argon
Nebulizer gas flow	0.8 L/min argon
Sample uptake rate	1.5 mL/min
Viewing mode	Axial view
Replicate readings	3
Integration time	3 to 5s
Rinse time	30s
Emission wavelength, Cr	267.716 nm
Emission wavelength, Mn	257.610 nm
Emission wavelength, Ni	231.604 nm
Emission wavelength, Cu	324.754 nm
Emission wavelength, Zn	213.857 nm
Emission wavelength, As	188.979 nm
Emission wavelength, Cd	228.802 nm
Emission wavelength, Pb	220.353 nm

Calibration used six calibration points plus a blank, prepared from multi-element standards traceable to the National Institute of Standards and Technology (NIST) and manufactured under ISO/IEC 17025 and ISO 17034 quality management systems, with calibration accepted at a coefficient of determination of 0.999 or better. Continuing calibration verification standards were analyzed approximately every 10 to 20 samples and accepted within 90 to 110% of the expected value. Quality assurance included the certified reference material NIST SRM 2711a, digested and analyzed with each batch, for which recoveries ranged from 94.8 to 103.2% against a laboratory acceptance window of 90 to 110%. Method blanks, laboratory duplicates and matrix spikes were also analyzed. Because Method 3050B is a partial digestion that recovers the environmentally available fraction rather than total elemental content, the ICP-OES results are not directly equivalent to the pXRF measurements and were not used to construct or correct a site-specific pXRF calibration. The comparison in Sect. “Comparison of pXRF with laboratory ICP-OES” is therefore an independent method comparison between two different operational fractions, not a validation.

### Statistical analysis and risk assessment

Analyses were performed in R. Relationships among elements were examined by Pearson correlation, with Spearman rank correlation used where distributions were strongly skewed or influenced by outliers. Source groups were identified by principal component analysis on the 16 depth-specific element variables (seven elements at two depths plus total iron at two depths), computed on centered and scaled data, with components retained where the eigenvalue exceeded unity. Hierarchical cluster analysis was applied to the same variables using one minus the absolute correlation as the distance measure and average linkage. Association between waste composition and elemental burden was tested by Mann–Whitney tests comparing sites where each waste category was present against sites where it was absent; these tests are exploratory, since with 45 sites, presence-absence coding, unbalanced groups and seven categories the analysis has limited power and substantial multiplicity.

A screening-level human health risk assessment was carried out following U.S. EPA guidance for residential exposure (U.S. Environmental Protection Agency, 1989), with exposure parameters taken from the Exposure Factors Handbook (U.S. Environmental Protection Agency, 2011), evaluating incidental soil ingestion, dermal contact and inhalation of resuspended particulates for a child and an adult resident. Toxicity values, dermal absorption fractions where available, and residential soil screening levels were taken from the current U.S. EPA Regional Screening Level tables (U.S. Environmental Protection Agency, 2026). Hazard quotients were calculated as the ratio of the estimated dose to the reference dose, and summed across elements to a hazard index. Incremental lifetime cancer risk was calculated for arsenic using the oral slope factor. Because the pXRF determines total chromium and does not resolve speciation, chromium was evaluated using the Cr(III) reference dose, and a sensitivity analysis was run assuming the whole of the measured chromium to be Cr(VI). Lead was not evaluated by hazard quotient, because EPA assigns lead no reference dose and evaluates it through blood-lead modeling; lead was instead compared against the residential soil screening level of 200 mg/kg and the removal management level of 600 mg/kg (U.S. Environmental Protection Agency, 2025). Dermal absorption fractions are supplied by EPA for arsenic and cadmium only; for the remaining elements a literature default of 0.001 was applied, and this is identified where the results are reported.

## Results

### Site characteristics by ward

Table 5 summarizes the distribution and characteristics of cataloged illegal dumping sites by ward. Forty-five sites were identified across Jackson. No sites were cataloged in Ward 1; this reflects the absence of reported dumping there during the study period and not the use of Ward 1 as a control, and no reference or uncontaminated control sites were sampled in this study. Ward 5 contained the largest number of sites (n = 15) and Ward 4 the fewest (n = 2). Ward 2 had the largest mean site perimeter at 334 m, but its median perimeter was 200 m, and the same right-skew is present in most wards, so both statistics are reported. Across all sites the most frequently recorded materials were domestic waste (41 sites), tires (38), construction debris (33) and furniture (31). Chemicals were recorded at 21 sites, electronic waste at 14 and yard waste at 6. Because categories were recorded as present or absent, these counts describe how often a material was observed and not how much of it was present.

**Table 5:** Characteristics of cataloged illegal dumping sites by ward

Number of sites and size	Ward 1	Ward 2	Ward 3	Ward 4	Ward 5	Ward 6	Ward 7	Total
Illegal dumping sites cataloged	0	8	6	2	15	7	7	45
Mean perimeter (m)	0	334.38	143.73	194.5	138.16	210.35	172.85	–
Median perimeter (m)	–	200	83.7	194	108	191	112	–
*Sites by contents*								
Tires	0	6	5	1	13	7	6	38
Furniture	0	5	3	1	11	4	7	31
Domestic waste	0	8	5	2	14	6	6	41
Construction debris	0	7	3	2	9	7	5	33
Chemicals	0	1	2	1	9	5	3	21
Electronic waste (e-waste)	0	1	2	1	5	2	3	14
Yard waste	0	0	0	0	3	0	3	6

### Soil concentrations of potentially toxic elements by ward

**Fig. 2:**
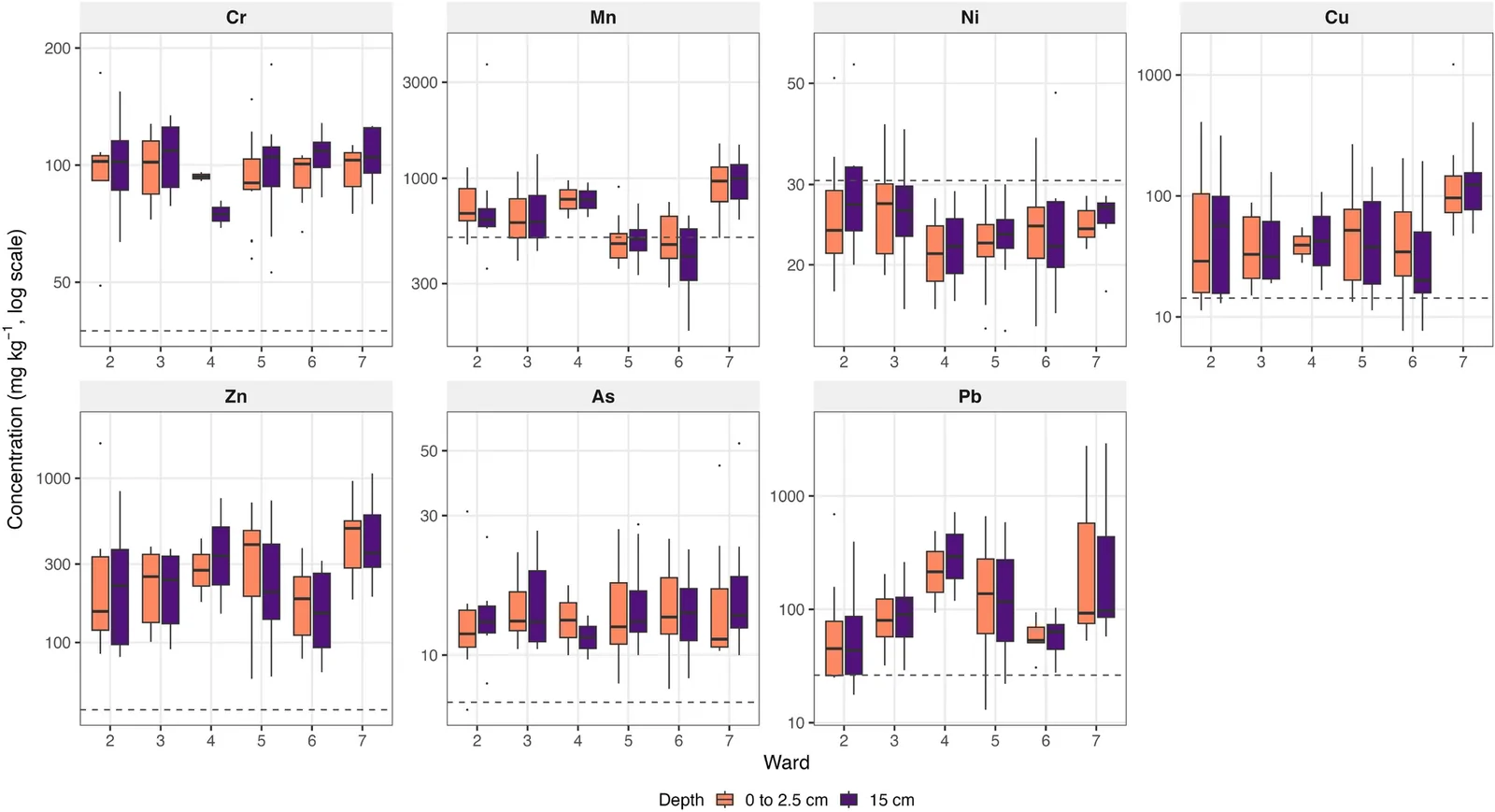
Distributions of potentially toxic element concentrations by ward and depth across 45 illegal dumping sites, shown on a logarithmic scale. Boxes give the median and interquartile range and whiskers extend to 1.5 times the interquartile range; points beyond that are plotted individually. The dashed line in each panel marks the local background value for that element (Table 1)

Supplementary Table S2 reports concentrations by ward and depth, and Fig. 2 shows the distributions with each element's local background marked. Concentrations were broadly similar between the two depths. Across all 45 sites, surface and 15 cm medians were 100 and 104 mg/kg for chromium, 602 and 590 mg/kg for manganese, 23.7 and 24.0 mg/kg for nickel, 47.7 and 44.0 mg/kg for copper, 252 and 260 mg/kg for zinc, 12.5 and 13.7 mg/kg for arsenic, and 84.7 and 78.7 mg/kg for lead. The distributions are strongly right-skewed for the waste-associated elements, so means substantially exceed medians: surface lead averaged 226 mg/kg against a median of 84.7 mg/kg, and surface copper averaged 100 mg/kg against a median of 47.7 mg/kg. Medians are therefore reported alongside means throughout, and ward-level means are interpreted with the influence of individual extreme sites made explicit. Maximum concentrations were high and site-specific, reaching 2770 mg/kg for lead at the surface and 2910 mg/kg at 15 cm, 1630 mg/kg for zinc, 1220 mg/kg for copper and 3680 mg/kg for manganese at 15 cm. Cadmium was above the pXRF reporting threshold at only one of 45 sites at the surface, at 18 mg/kg, and at three of 45 sites at 15 cm, at 19, 24 and 27 mg/kg; all remaining cadmium observations were non-detects. Every other element was detected at all 45 sites at both depths. Total iron averaged 20,900 mg/kg at the surface (range 10,700 to 32,900 mg/kg) and 21,500 mg/kg at 15 cm.

Soil pH, total iron and texture by ward are given in Table 6. Soil pH ranged from 3.0 to 8.7 across the 45 sites, with 28 sites acidic, 12 near neutral and 5 alkaline. Surface texture was silt loam at 43 of 45 sites, and at 15 cm the profile was silt loam at 31 sites and silty clay loam at 13. Sites were developed predominantly on Loring, Byram, Siwell and Riedtown map units; the full distribution of soil series is given in Supplementary Table S7.

**Table 6:** Soil properties of illegal dumping sites by ward

Ward	n	pH mean	pH min	pH max	Fe 0 to 2.5 cm (mg/kg)	Fe 15 cm (mg/kg)
2	8	5.82	3.7	8.0	21,100	21,900
3	6	5.17	3.0	6.9	22,200	23,000
4	2	7.60	6.5	8.7	18,100	18,300
5	15	5.81	3.2	8.1	20,800	21,200
6	7	5.10	3.0	7.0	20,900	21,900
7	7	6.29	3.9	8.3	20,600	21,000

pH was measured in the field with a probe. Total iron was determined by portable XRF. Surface texture was silt loam at 43 of 45 sites; at 15 cm the profile was silt loam at 31 sites and silty clay loam at 13. Soil series and texture are survey-derived attributes and were not determined on the collected samples

### Element-specific enrichment: contamination factor

**Fig. 3:**
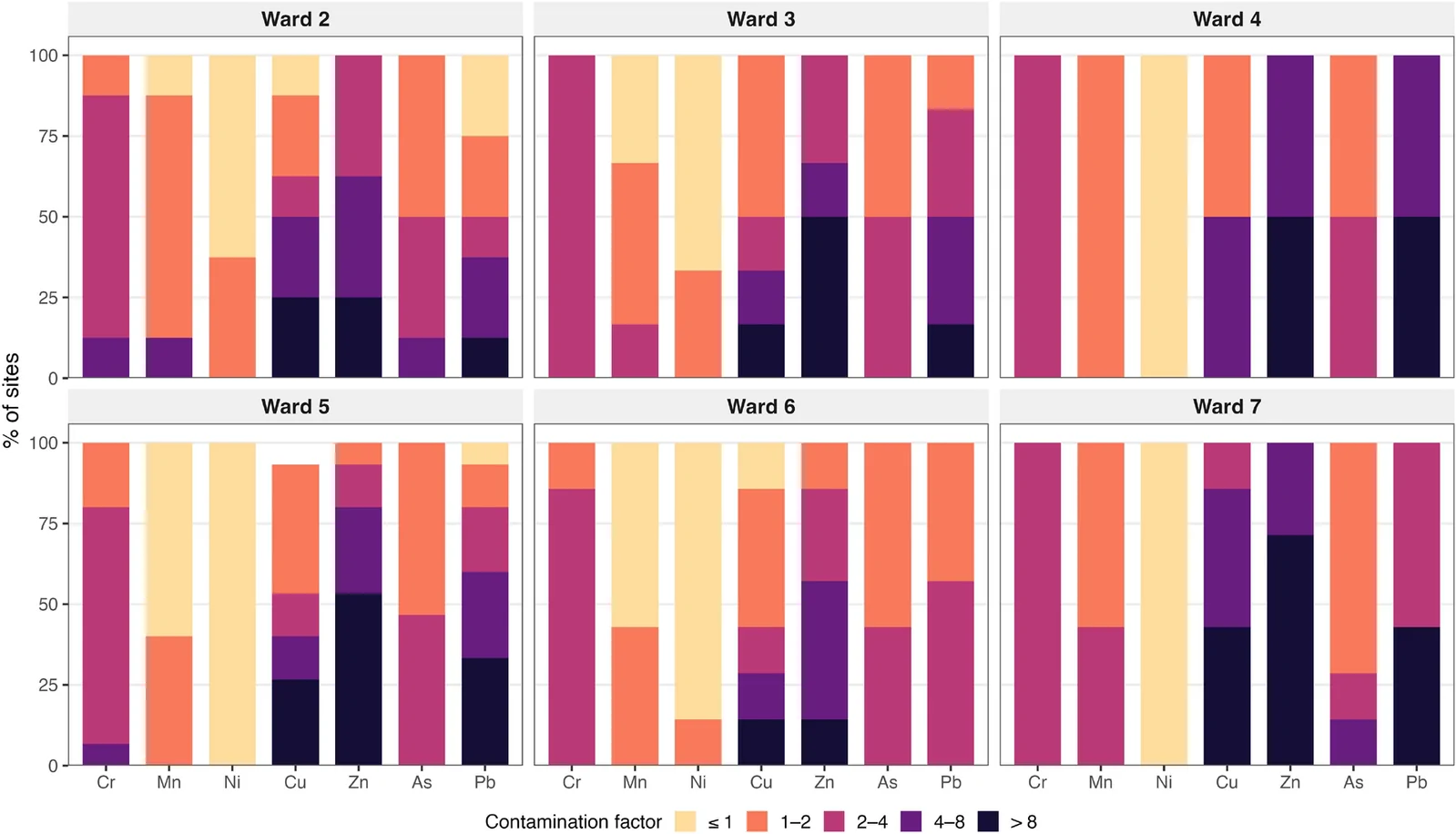
Distribution of single-element contamination factor (Cf) classes across illegal dumping sites, by ward and element

Supplementary Table S3 summarizes contamination factor (Cf) values by ward, and Fig. 3 shows the distribution of Cf pollution classes by ward and element. Cf values were unevenly distributed and elements contributed differently to ward-level patterns. Mean Cf for chromium was similar across all wards (2.25 to 2.77) and mean Cf for nickel was below unity everywhere (0.73 to 0.94), indicating no enrichment above background for nickel. Ward 7 had the highest mean Cf for manganese (1.94), copper (14.4), zinc (12.3), arsenic (2.72) and lead (22.8), the strongest overall single-element burden among the sampled wards. These ward-level patterns are consistent with the mixed-waste inventory in Table 5: in Ward 7 most sites contained tires (6 of 7), furniture (7 of 7), domestic waste (6 of 7) and construction debris (5 of 7), with chemicals, electronic waste and yard waste each recorded at 3 of 7 sites. This mixture provides plausible source material for the lead, copper and zinc enrichment observed, while the exploratory tests reported in Sect. “Element associations and source groups” show that visible waste categories are weak predictors of measured burden and should not be treated as substitutes for soil measurement.

Maximum Cf values identified element-specific hotspots. The highest single-site Cf values were for lead in Ward 7, at 107, and for zinc in Ward 2, at 33.4. Overall, lead, copper and zinc were the primary pollutants across the 45 sites, whereas chromium, manganese, nickel and arsenic contributed less: mean Cf across wards remained between 2.25 and 2.77 for chromium, 0.93 and 1.94 for manganese, 0.73 and 0.94 for nickel, and 1.84 and 2.72 for arsenic. Because these indices are referenced to regional background values whose limitations are set out in Sect. “Composite contamination indices”, they are used here to rank and compare sites rather than to characterize hazard.

Across Wards 2 through 7, zinc, copper and lead were the elements most often in the severe and very severe pollution categories. Ward 7 had the highest proportion of sites with very severe zinc pollution (71.4%) and showed very severe copper and lead pollution at 42.9% of sites each. Ward 5 also showed widespread zinc and lead pollution, with 53.3% of sites very severe for zinc and 33.3% very severe for lead. Zinc was very severely enriched at half the sites in Wards 3 and 4 as well, and lead at half the sites in Ward 4. Ward 6 showed the lowest proportions, with 14.3% of sites very severe for copper and for zinc and none for lead.

Each panel corresponds to one Jackson ward (Wards 2 to 7; n = 8, 6, 2, 15, 7 and 7 sites respectively; Ward 1 contained no cataloged sites). Within a panel the seven bars represent the seven elements retained in the indices (Cr, Mn, Ni, Cu, Zn, As, Pb). Cadmium is not shown, because the portable XRF cadmium readings are not corroborated by ICP-OES and the element is excluded from the contamination factor and the degree of contamination (Sects. “Composite contamination indices” and “Comparison of pXRF with laboratory ICP-OES”). The height of each colored segment is the percentage of that ward's sites whose Cf for that element falls in the indicated range. Cf is the ratio of the measured soil concentration, the mean of the 0 to 2.5 cm and 15 cm composite samples by portable XRF, to the local background value in Table 1. A Cf at or below 1 indicates no enrichment above background; the pollution classes are slight (1 to 2), moderate (2 to 4), severe (4 to 8) and very severe (above 8). Bars sum to 100% within each ward.

### Cumulative burden: degree of contamination

**Fig. 4:**
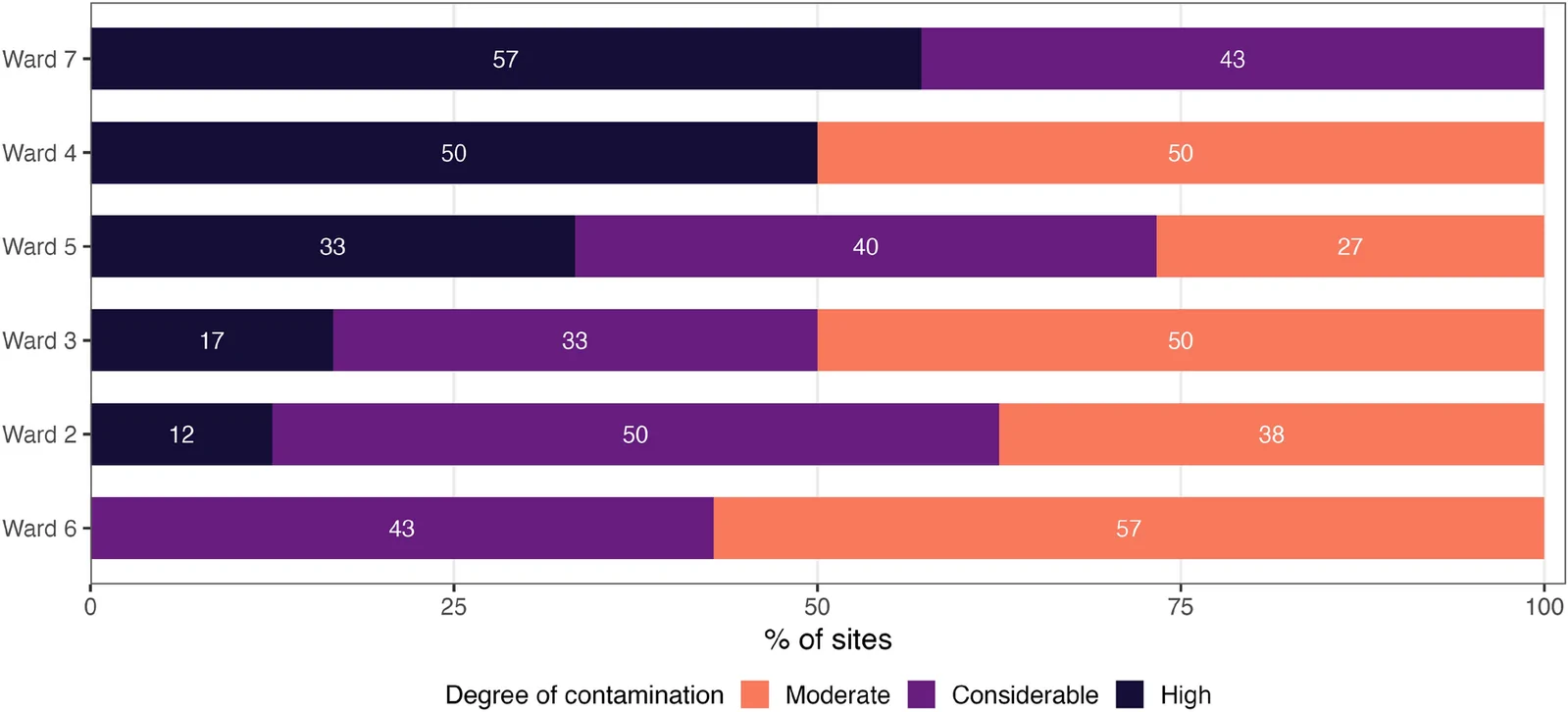
Distribution of the degree of contamination (Cdeg) across illegal dumping sites, by ward

The degree of contamination, which sums the single-element Cf values into a cumulative measure, was highest in Ward 7 by mean and lowest in Ward 6. Ward-level means were 57.7 for Ward 7, 33.3 for Ward 4, 27.3 for Ward 5, 26.8 for Ward 2, 20.9 for Ward 3 and 17.8 for Ward 6. Medians tell a materially different story and are reported alongside: 33.5 for Ward 7, 33.3 for Ward 4, 25.8 for Ward 5, 21.2 for Ward 2, 19.8 for Ward 3 and 15.6 for Ward 6. By median, Ward 7 and Ward 4 are therefore effectively indistinguishable, and Ward 7’s high mean reflects the presence of the most extreme sites in the dataset rather than a uniformly elevated ward: its maximum Cdeg was 153, against 50.7 in Ward 4. Lead, copper and zinc were the main contributors to the cumulative burden. Across the city, no site fell in the low class, 15 sites were moderate, 18 considerable and 12 high. Ward 7 had the largest proportion of high-class sites (4 of 7) and Ward 6 none (Fig. 4). Including cadmium in an eight-element index changes the classification of two sites and leaves the ward ranking unaltered (Supplementary Table S3), so the cumulative pattern does not depend on that choice.

Each bar represents one Jackson ward (Wards 2 to 7; n = 8, 6, 2, 15, 7, and 7 sites) and is divided into the proportion of that ward’s sites in each Cdeg class; bars are ordered from the highest to the lowest percentage of sites in the most contaminated class. Cdeg is the sum of the seven single-element contamination factors at a site and is classified after Hakanson (1980) as low (Cdeg below 8), moderate (8 to 16), considerable (16 to 32), or high (32 or above). Shading darkens with increasing contamination, and bars sum to 100% within each ward. Under the seven-element index, 15 sites are moderate, 18 considerable and 12 high, and no site falls in the low class. Ward 7 has the largest proportion of high-class sites (4 of 7) and Ward 6 none; Ward 4, with only two sites, has one moderate and one high. Because the distributions are strongly right-skewed, medians are reported alongside means in the text.

### Relationships among elements and across depth

**Fig. 5:**
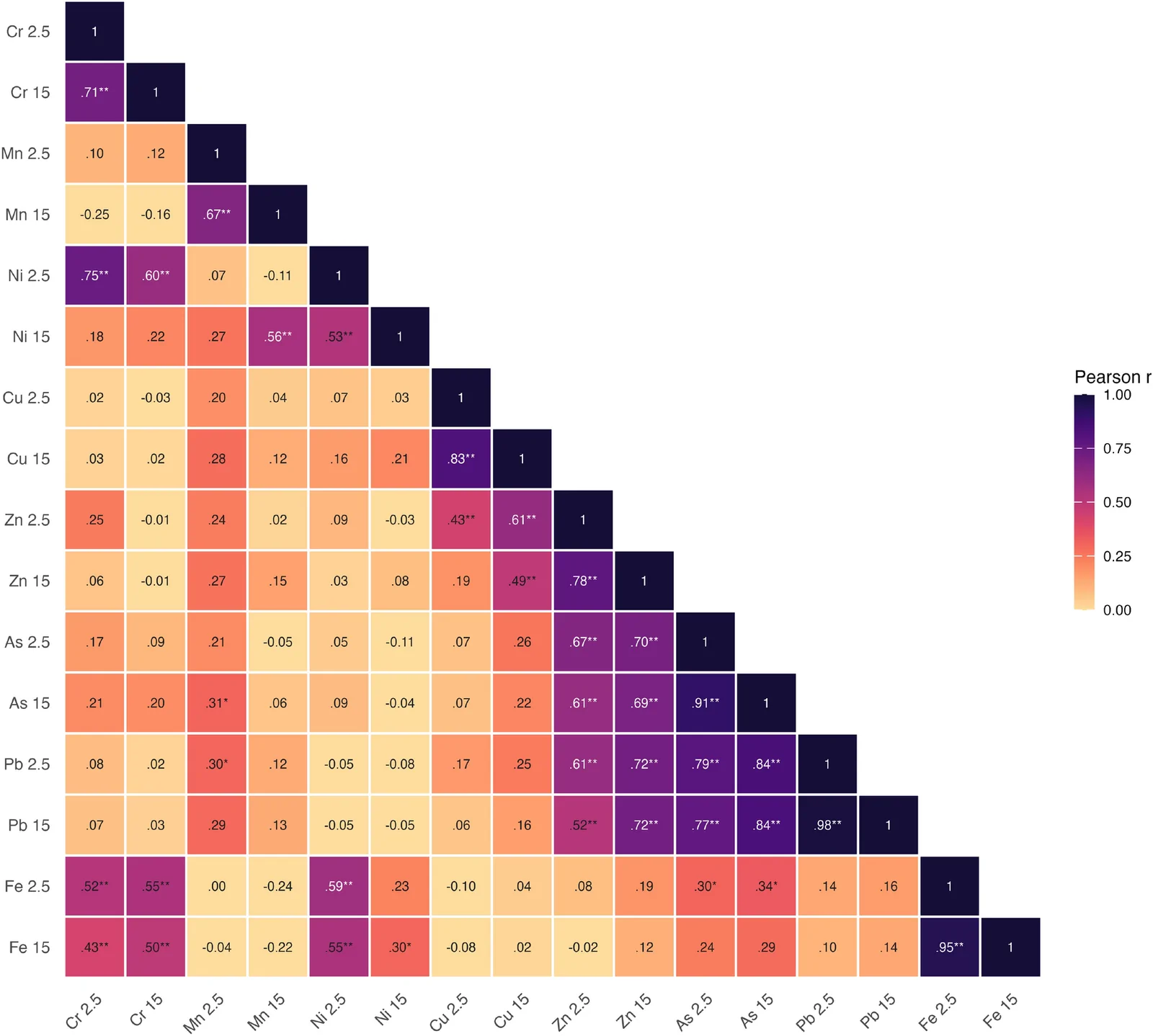
Pearson correlation matrix of soil concentrations of potentially toxic elements measured by portable XRF across 45 illegal dumping sites in Jackson, Mississippi, with each element shown at both depths and total iron included. Variable labels give the element followed by the sampling depth in centimetres, so “Cr 2.5” is chromium at 0 to 2.5 cm and “Cr 15” is chromium at 15 cm. Only the lower triangle is shown. Cell colour and value give the correlation coefficient (r); asterisks denote significance (* *p* < 0.05, ** *p* < 0.01) and the diagonal shows the self-correlation. Cadmium is excluded because too few sites had detectable concentrations. Strong within-element correlations across depths indicate vertically consistent enrichment; iron co-varies with chromium and nickel but not with copper, zinc or lead

Correlation analyses showed strong relationships among elements across the sites (Fig. 5). Surface and 15 cm concentrations were strongly correlated for every element: lead (r = 0.982, *p* < 0.001), arsenic (r = 0.907, *p* < 0.001), copper (r = 0.825, *p* < 0.001), zinc (r = 0.782, *p* < 0.001), chromium (r = 0.715, *p* < 0.001), manganese (r = 0.670, *p* < 0.001) and nickel (r = 0.531, *p* < 0.001). Several cross-element correlations were also identified. Arsenic and lead were strongly correlated across depths, including arsenic at 15 cm with lead at the surface (r = 0.842, *p* < 0.001) and at 15 cm (r = 0.836, *p* < 0.001). Zinc was positively correlated with both lead and arsenic, including zinc at 15 cm with lead at the surface (r = 0.718, *p* < 0.001) and arsenic at the surface (r = 0.702, *p* < 0.001). Chromium and nickel were correlated with one another at the surface (r = 0.747, *p* < 0.001) but not with the lead, arsenic and zinc group. Cadmium was excluded from the correlation analysis because it was detected at too few sites.

Total iron was included to test whether the measured elements co-vary with iron-bearing phases. Chromium and nickel did: chromium correlated with surface iron at r = 0.520 (*p* < 0.001) and 15 cm chromium with surface iron at r = 0.547 (*p* < 0.001), while surface nickel correlated with surface iron at r = 0.585 (*p* < 0.001). Arsenic showed a weaker but significant association (r = 0.304, *p* = 0.043 at the surface; r = 0.338, *p* = 0.023 for 15 cm arsenic with surface iron). Copper, zinc and lead showed no association with iron at either depth, with all coefficients between negative 0.10 and 0.19 and none significant. Manganese also showed no association with iron (r = 0.000 to negative 0.24, none significant), which is relevant because manganese oxides are frequently invoked as a binding phase for trace elements; in these soils manganese does not co-vary with iron and behaves independently of both element groups. Full iron correlations with significance levels are given in Supplementary Table S6.

### Element associations and source groups

Principal component analysis of the 16 depth-specific variables retained four components with eigenvalues above unity, together explaining 79.8% of the variance (Table 7). The first component (33.4%) was loaded by zinc, arsenic and lead at both depths (loadings 0.335 to 0.387), with copper contributing at 15 cm (0.210). The second component (21.9%) was loaded by chromium, nickel and iron (0.385 to 0.429 for chromium and nickel at the surface, 0.402 and 0.407 for iron), and carried essentially no loading from lead, zinc or arsenic. The third component (13.8%) was loaded by manganese together with 15 cm nickel and copper, and the fourth (10.6%) contrasted manganese against copper.

**Table 7:** Principal component loadings for the 16 depth-specific variables

Variable	PC1	PC2	PC3	PC4
Cr, 0 to 2.5 cm	0.152	0.385	0.021	−0.105
Cr, 15 cm	0.117	0.398	0.033	0.009
Mn, 0 to 2.5 cm	0.173	−0.077	0.373	0.313
Mn, 15 cm	0.044	−0.166	0.447	0.480
Ni, 0 to 2.5 cm	0.118	0.429	0.189	−0.060
Ni, 15 cm	0.054	0.194	0.457	0.277
Cu, 0 to 2.5 cm	0.124	−0.108	0.352	−0.506
Cu, 15 cm	0.210	−0.089	0.386	−0.429
Zn, 0 to 2.5 cm	0.335	−0.133	0.068	−0.269
Zn, 15 cm	0.359	−0.140	0.005	−0.019
As, 0 to 2.5 cm	0.373	−0.074	−0.206	0.031
As, 15 cm	0.387	−0.048	−0.168	0.123
Pb, 0 to 2.5 cm	0.371	−0.161	−0.140	0.111
Pb, 15 cm	0.359	−0.143	−0.171	0.188
Fe, 0 to 2.5 cm	0.185	0.407	−0.102	0.030
Fe, 15 cm	0.156	0.402	−0.086	0.055
Eigenvalue	5.352	3.503	2.211	1.704
Variance explained (%)	33.4	21.9	13.8	10.6
Cumulative variance (%)	33.4	55.3	69.2	79.8

Contamination factors are calculated for the seven elements detected at every site and both depths, so no non-detect substitution is applied. Cadmium is excluded and reported descriptively in Sect. “Soil concentrations of potentially toxic elements by ward”. An eight-element calculation including cadmium is given in Supplementary Table S3 as a sensitivity analysis

**Fig. 6:**
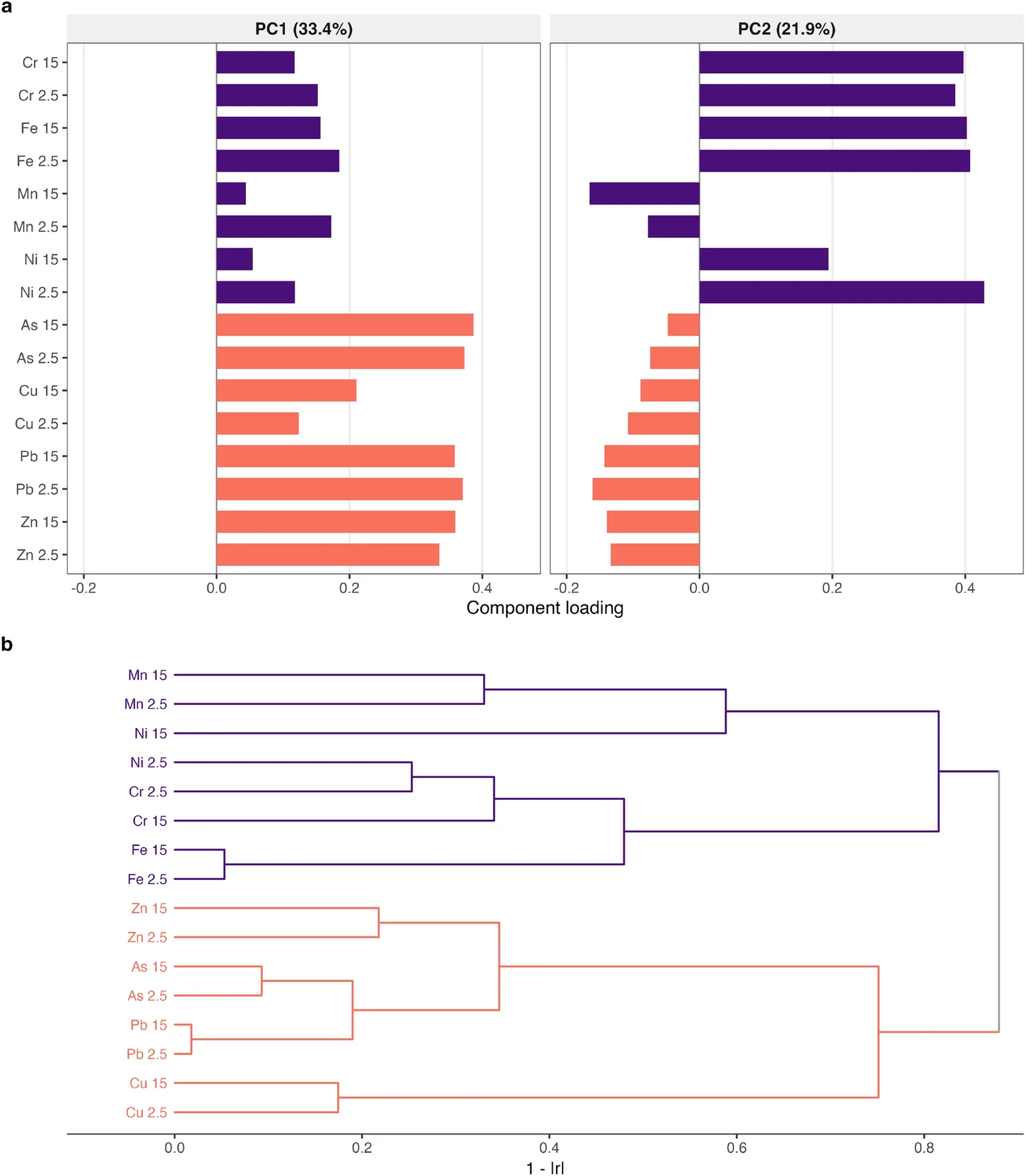
Element associations across 45 illegal dumping sites. Variable labels give the element followed by the sampling depth in centimetres. **a** Principal component loadings of the 16 depth-specific variables on the first two components, with bars coloured by the two-cluster grouping shown in panel b and the percentage of variance explained given in each facet heading. **b** Hierarchical clustering of the same variables using one minus the absolute correlation as the distance measure and average linkage, with branches coloured by the two-cluster cut, which separates copper, lead, arsenic and zinc from iron, chromium, nickel and manganese

Hierarchical cluster analysis of the same variables recovered the same structure independently (Fig. 6). At a two-cluster cut, one cluster contained copper, lead, arsenic and zinc and the other contained iron, chromium, nickel and manganese. Within the dendrogram every element joined its own counterpart at the other depth before joining any other element, which is direct structural evidence for the vertical coherence indicated by the depth correlations in Sect. “Relationships among elements and across depth”.

**Fig. 7:**
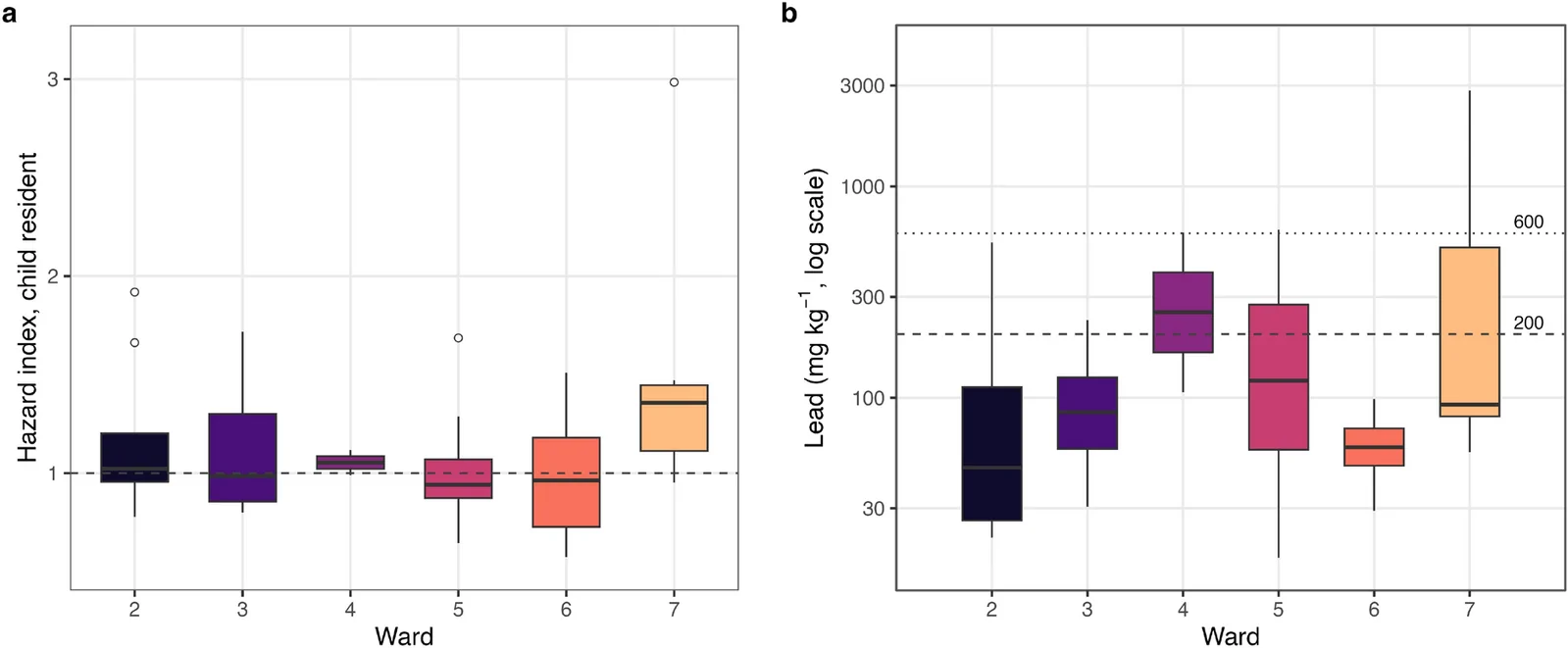
Screening-level health indicators by ward. **a** Hazard index for a child resident, with the dashed line at unity. **b** Depth-averaged soil lead concentration on a logarithmic scale, with the United States Environmental Protection Agency residential soil screening level of 200 mg/kg and removal management level of 600 mg/kg marked

Taken with the iron correlations and with the partial-extraction recoveries reported in Sect. “Comparison of pXRF with laboratory ICP-OES”, these analyses support a two-source interpretation. Chromium and nickel co-vary with iron and are recovered only weakly by partial acid extraction, which is consistent with a lithogenic association in iron-bearing and silicate phases. Zinc, copper and lead do not co-vary with iron and are recovered more readily by partial extraction, which is consistent with waste-derived inputs. Arsenic loads with the waste-associated group but also shows a weak iron association, consistent with both an anthropogenic contribution and sorption to iron oxyhydroxides. Manganese behaves independently of both groups and is a rock-forming element rather than a contaminant characteristic of dumped material (Fig. 7).

Association between visible waste composition and measured burden was weak. None of the seven waste categories predicted the first component or the degree of contamination (Mann–Whitney, all *p* > 0.19). Chemical waste was associated with the second component (*p* = 0.007) and with surface chromium (*p* = 0.016). With 45 sites, presence-absence coding, unbalanced group sizes and seven categories tested, these results are exploratory and would not survive strict correction for multiplicity. The practical implication is that visible waste categories are poor predictors of subsurface elemental burden and cannot substitute for measurement.

### Comparison of pXRF with laboratory ICP-OES

ICP-OES results were compared with pXRF measurements on the paired subset to assess how the two techniques relate (Table 8; n = 10 at the surface and 13 at 15 cm, reflecting sample availability). Correlations between the techniques were strong for lead (r = 0.990 at the surface and 0.991 at 15 cm), copper (0.987 and 0.953) and zinc (0.883 and 0.865), moderate for chromium (0.852 and 0.729) and arsenic (0.557 and 0.710), and weak for nickel (0.472 and 0.484). Manganese was strongly correlated at the surface (0.914) but not at depth (0.357).

**Table 8:** Comparison of portable XRF with laboratory ICP-OES on the paired subset

Element	Depth (cm)	n	pXRF mean (mg/kg)	ICP-OES mean (mg/kg)	Median ICP/pXRF ratio	Pearson r	Cf class agreement (%)
Cr	0 to 2.5	10	79.6	7.6	0.09	0.852	0
Cr	15	13	94.3	10.1	0.11	0.729	
Mn	0 to 2.5	10	646	512	0.86	0.914	69
Mn	15	13	844	323	0.48	0.357	
Ni	0 to 2.5	10	21.8	6.8	0.31	0.472	85
Ni	15	13	27.3	17.5	0.41	0.484	
Cu	0 to 2.5	10	170	114	0.60	0.987	39
Cu	15	13	98.4	51.1	0.54	0.953	
Zn	0 to 2.5	10	308	126	0.39	0.883	8
Zn	15	13	283	112	0.44	0.865	
As	0 to 2.5	10	14.9	3.8	0.26	0.557	15
As	15	13	15.8	4.3	0.29	0.710	
Pb	0 to 2.5	10	222	108	0.51	0.990	62
Pb	15	13	162	83.6	0.53	0.991	

EPA Method 3050B is a partial extraction recovering the environmentally available fraction, whereas portable XRF measures total elemental content, so ratios below unity are expected. Cf class agreement is the percentage of paired sites placed in the same contamination factor class by both techniques. Agreement in degree of contamination class was 1 of 13 sites (Cohen’s kappa = −0.061)

Absolute concentrations differed systematically, with ICP-OES consistently lower. Median ratios of ICP-OES to pXRF concentration were 0.09 and 0.11 for chromium, 0.26 and 0.29 for arsenic, 0.31 and 0.41 for nickel, 0.39 and 0.44 for zinc, 0.51 and 0.53 for lead, 0.60 and 0.54 for copper, and 0.86 and 0.48 for manganese. This offset is expected rather than an analytical discrepancy: Method 3050B is a nitric acid and peroxide extraction that recovers the environmentally available fraction, whereas pXRF measures total elemental content, so the two quantify different operational pools. The magnitude of the offset is itself informative. Chromium recovered at roughly 10% of the total is consistent with chromium held in silicate phases that nitric acid does not liberate, which supports the lithogenic assignment of chromium in Sect. “Element associations and source groups”, whereas lead and copper recovered at about half of total are consistent with more readily extractable, waste-derived forms.

Because the two techniques measure different fractions, contamination indices calculated from each do not agree. Computing the degree of contamination from both datasets on the paired subset, the classification matched for only 1 of 13 sites, with Cohen's kappa of negative 0.061, and ICP-OES placed sites in a lower class in every case of disagreement. Agreement in single-element Cf class ranged from 85% for nickel and 69% for manganese down to 62% for lead, 39% for copper, 15% for arsenic, 8% for zinc and none for chromium. Index-based classification is therefore method-dependent, and the classifications reported in this paper are explicitly those obtained from total concentrations by pXRF. This dependence is a further reason to treat Cf and Cdeg as screening and prioritization devices rather than as measurements of contamination status.

Cadmium requires separate comment, because the two techniques disagree in kind rather than in degree. Portable XRF placed cadmium above its reporting threshold at only four of the 45 sites, at 18 to 27 mg/kg, whereas ICP-OES detected cadmium in all 13 paired samples at 15 cm and in 10 of 13 at the surface, at concentrations of 0.047 to 0.638 mg/kg. The laboratory values are 30 to 570 times lower than the four pXRF readings and lie below the local background of 1.0 mg/kg. We therefore treat the pXRF cadmium readings as spectral artifacts rather than measurements, and the ICP-OES data as showing cadmium to be present throughout these soils at concentrations well below the EPA residential soil screening level of 7.1 mg/kg, which the highest laboratory value reaches only 9% of. Cadmium was therefore detected by the laboratory method, but at concentrations portable XRF cannot resolve. This is the basis for excluding cadmium from the contamination indices in Sect. “Composite contamination indices” and from the hazard index in Sect. “Screening-level human health risk”.

### Screening-level human health risk

Hazard quotients and hazard indices for a child and an adult resident are summarized by ward in Table 9. Cadmium is excluded from the index for the reason given in Sect. “Comparison of pXRF with laboratory ICP-OES” and is considered separately below. For a child resident the hazard index exceeded unity at 21 of 45 sites, with a mean of 1.12, a median of 0.990 and a maximum of 2.98. The index was dominated by two elements: arsenic, with a mean hazard quotient of 0.699 and 7 sites above unity, and manganese, with a mean of 0.363 and one site above unity. Copper, zinc, nickel and chromium contributed negligibly, with mean hazard quotients of 0.028, 0.014, 0.016 and 0.001 respectively. Excluding arsenic, the hazard index exceeded unity at only one of 45 sites and the median fell to 0.365. For an adult resident the hazard index remained below unity at every site, with a maximum of 0.291, so the exposure concern identified here is specific to young children.

**Table 9:** Screening-level health indicators by ward

Ward	n	HI child, mean	HI child, median	Sites HI > 1	HI child excl. As, median	HI adult, max	ILCR child, max	Pb median (mg/kg)	Pb max (mg/kg)	Sites Pb > 200
2	8	1.17	1.02	5	0.401	0.186	4.6 × 10^−5^	48.8	543	1
3	6	1.12	0.986	2	0.376	0.166	3.6 × 10^−5^	90.1	233	1
4	2	1.05	1.05	1	0.476	0.108	2.6 × 10^−5^	356	606	1
5	15	1.00	0.941	5	0.316	0.164	4.5 × 10^−5^	120	625	5
6	7	0.980	0.963	2	0.327	0.147	4.0 × 10^−5^	58.3	98.7	0

HI, hazard index; ILCR, incremental lifetime cancer risk from arsenic by soil ingestion. Lead concentrations are depth-averaged. Lead is compared against the EPA residential soil screening level of 200 mg/kg rather than evaluated by hazard quotient, because EPA assigns lead no reference dose. Chromium was evaluated using the Cr(III) reference dose; see Sect. “Screening-level human health risk” for the Cr(VI) sensitivity analysis. Cadmium is excluded from the hazard index because the portable XRF cadmium readings are not corroborated by ICP-OES; see Sect. “Comparison of pXRF with laboratory ICP-OES”.

Incremental lifetime cancer risk from arsenic by soil ingestion averaged 2.5 × 10^−5^ for a child resident, with a maximum of 8.0 × 10^−5^. All 45 sites exceeded 10^−5^ and none exceeded 10⁻4, so estimated cancer risk lies within the range EPA regards as acceptable but above the point of departure.

Two features of these results require care in interpretation. First, the elements that dominate the calculated hazard are largely geogenic in this setting. The EPA residential screening level for arsenic is 0.68 mg/kg, which is below the local background of 6.9 mg/kg, and EPA guidance notes that arsenic and manganese commonly exceed risk-based screening levels at natural background concentrations. Arsenic accordingly exceeded its screening level at all 45 sites, and that universal exceedance is not evidence of a dumping impact. Second, chromium was evaluated using the Cr(III) reference dose because speciation was not determined. This assumption matters: if the whole of the measured chromium were Cr(VI), the mean child hazard quotient for chromium by ingestion alone would rise from 0.001 to 1.42, and even a 1% Cr(VI) fraction would give 0.014. Chromium hazard is therefore unresolved rather than negligible, and speciation would be required to settle it.

Lead was assessed separately, because EPA assigns lead no reference dose and evaluates it through blood-lead modeling. Compared against the residential soil screening level of 200 mg/kg, depth-averaged lead exceeded that level at 11 of 45 sites (24%), and exceeded the 600 mg/kg removal management level at 4 sites (9%). Exceedances were concentrated but not confined to particular wards: 5 of 15 sites in Ward 5, 3 of 7 in Ward 7, and one site each in Wards 2, 3 and 4 exceeded 200 mg/kg, while no site in Ward 6 did. This comparison requires no background value and no index, and is the most defensible health-relevant statement the dataset supports. Of the other elements, manganese exceeded its screening level of 1,800 mg/kg at one site; chromium, nickel, copper, zinc and cadmium remained below their screening levels at every site.

## Discussion

### Illegal dumping as a source of disturbed urban soil chemistry

The central finding of this study is that illegal dumping leaves a measurable elemental signature in soil that extends below the visible surface. Because no waste was removed by the study team, the sites were characterized as they stood, with waste present, and the depth data therefore describe what is already in the ground beneath an active dumping site rather than what remains after a cleanup. The practical inference is the same and is the more important one: because concentrations at 15 cm approach those at the surface, removing the surface waste, or even the uppermost soil, would not remove the elemental burden. Most of the cataloged sites held far more than household refuse; tires, construction and demolition debris, chemicals, electronics and furniture were common, and these materials are recognized sources of the elements measured here (Vongdala et al., 2019; Wuana & Okieimen, 2011).

The element patterns we measured are consistent with the materials present at the sites. Lead, copper, and zinc, which contributed most to the overall burden, are characteristic of mixed urban waste streams, including construction and demolition debris, electronic waste, discarded tires, and painted or treated materials (Wuana & Okieimen, 2011). The co-occurrence of these elements with visibly mixed waste suggests that dumping sites behave as unmanaged mixed-waste environments rather than as simple accumulations of trash. Viewed this way, illegal dumping sits at the start of a source-to-receptor sequence: the waste is the source, the soil is the medium that stores and transforms the contaminants, and nearby residents and future site users are the potential receptors.

### The geochemical signature: dominant elements and cumulative burden

Across the 45 sites the contamination indices describe an uneven signature in which lead, copper and zinc are the dominant single-element contributors, while chromium, manganese and arsenic are modestly enriched and nickel is not enriched at all. The more substantive result, however, is that the elements separate into two groups on independent evidence. Principal component analysis and hierarchical clustering both place chromium, nickel and iron together and lead, zinc, arsenic and copper together, and the partial-extraction recoveries agree: chromium is recovered at roughly a tenth of its total concentration by nitric acid extraction, as expected for silicate-held chromium, whereas lead and copper are recovered at about half. Reading these three lines of evidence together allows a distinction that neither the indices nor single-element comparisons can make on their own, between elements present largely because of the local geology and elements present largely because of what was dumped.

Two cautions temper the interpretation. First, ward-level means are strongly influenced by individual sites. Ward 7 has the highest mean degree of contamination by a wide margin, but its median is indistinguishable from Ward 4's, and its mean lead concentration of 601 mg/kg sits against a median of 92.8 mg/kg because one site contains 2,838 mg/kg. Ward 7 is best described as containing the most extreme sites in the dataset rather than as a uniformly contaminated ward. Second, the indices quantify total element content referenced to regional background values, not the bioavailable fraction, and their classification depends on the analytical method used, as Sect. “Comparison of pXRF with laboratory ICP-OES” demonstrates. They are appropriate for ranking and prioritization and not for inference about dose or harm (Kowalska et al., 2018; Reimann & de Caritat, 2000).

### Environmental pathways: subsurface persistence and redistribution

The depth data point to enrichment that extends below the visible surface. For every element measured, surface and 15 cm concentrations were significantly correlated, and for lead the two depths were almost identical (r = 0.982). The cluster analysis reinforces this: each element paired with its own counterpart at the other depth before joining any other element, so vertical coherence appears as a property of the data structure and not only as a set of pairwise correlations. The direct practical implication is that surface clearance, or even removal of the uppermost soil layer, would not be sufficient to remove the elemental burden at these sites (Ihedioha et al., 2017; Kabata-Pendias, 2011).

The correlation structure indicates which elements travel together. Lead, arsenic and zinc were strongly correlated with one another across depths, a pattern consistent with shared sources in materials such as demolition debris, treated wood, painted surfaces, tires and scrap (Kabata-Pendias, 2011; Wuana & Okieimen, 2011). Chromium and nickel were correlated separately and with iron, consistent with a lithogenic association. Practically, this means a site flagged for elevated lead also warrants attention for arsenic, zinc and copper, which is the situation cumulative indices are designed to summarize.

We deliberately do not draw conclusions about element mobility. Mobility and bioavailability depend on parameters we did not determine on these samples, including laboratory-measured organic carbon, particle size distribution and the operationally defined fractions obtained by leaching or sequential extraction. Several transport processes could plausibly redistribute elements at and around dumping sites, including repeated dumping, vehicle traffic, weathering, soil disturbance and cleanup activity that mixes contaminated material downward, and flooding and stormwater can store and remobilise elements in urban soils and sediments (Ciszewski & Grygar, 2016; Huber et al., 2016). Jackson is drained by the Pearl River and its tributaries and several of the affected wards contain low-lying ground subject to periodic flooding, so these processes are plausible here. We did not measure them, and we treat them as inferred rather than demonstrated pathways; resolving their contribution would require repeated sampling and site hydrology beyond the scope of this cross-sectional study.

### Potential exposure and human health relevance

The clearest health-relevant statement the dataset supports concerns lead, and it rests on an absolute regulatory threshold rather than on any index or background value. EPA identifies 200 mg/kg as the residential soil screening level for lead and 600 mg/kg as the removal management level (U.S. EPA 2025). Depth-averaged lead exceeded 200 mg/kg at 11 of the 45 sites and 600 mg/kg at 4 sites, with a maximum of 2838 mg/kg. Exceedances occurred in five of the six sampled wards, most often in Ward 5, and lead is also the element that the component, cluster and extraction evidence identifies most clearly as waste-derived. The combination of a regulatory exceedance at nearly a quarter of sites and an unambiguous anthropogenic attribution is the principal public health finding of this study.

The screening-level risk assessment adds two things to that finding and qualifies a third. For a child resident the hazard index exceeded unity at 21 of 45 sites, whereas for an adult it remained below unity everywhere, so the exposure concern is specific to young children who have greater incidental soil ingestion relative to body weight. Estimated incremental lifetime cancer risk from arsenic averaged 2.5 × 10^−5^ and did not exceed 10^−4^ at any site, placing it within the range EPA treats as acceptable. The qualification is that the calculated hazard is driven by arsenic and manganese, and in this setting both are largely geogenic: the arsenic screening level of 0.68 mg/kg lies below the local background of 6.9 mg/kg, and EPA notes that arsenic and manganese commonly exceed risk-based levels at natural background. Removing arsenic from the index leaves only one of 45 sites above unity. The honest reading is therefore that the elements driving computed hazard are mostly natural, while the element driving regulatory exceedance is the one attributable to dumping. A study that had assumed dumping-derived risk without separating sources would have reached a different and less defensible conclusion.

These estimates describe screening-level potential exposure and not demonstrated harm. We did not measure bioaccessibility, plant uptake, resuspended dust or human exposure, and the cross-sectional design captures a single point in time. Chromium hazard is unresolved rather than negligible, because pXRF determines total chromium: under a Cr(III) assumption the child hazard quotient is 0.001, whereas if the whole of the measured chromium were Cr(VI) it would be 1.42. People may contact soil at these sites during cleanup, yard work, informal recreation, gardening or play, and fine particles can be resuspended as dust, but these pathways were not measured here. The health relevance is clearest where land use brings people into direct contact with soil: a former dumping site converted to a community garden, play area or housing-adjacent green space without soil assessment could expose residents, and children in particular, to elements that a visible cleanup did nothing to remove (Mitchell et al., 2014).

### Implications for remediation and environmental recovery

The spatial pattern has a direct management consequence. Contamination in Jackson is decentralized and site-specific: rather than radiating from a single landfill or industrial source, elevated element concentrations are scattered across individual dumping locations that differ in size, contents, and surroundings. Because the burden is uneven, a single uniform cleanup standard will not fit the city. The data instead support a site-by-site approach that separates locations needing only routine waste removal from those needing deeper soil assessment, confirmatory laboratory testing, remediation, or land-use restrictions. The pattern also suggests that future analyses should pair soil chemistry with built-environment indicators such as abandoned buildings, vacancy, housing age, demolition activity, and surrounding land use, because these factors may help explain why certain sites become element hotspots.

This is also where the distinction between cleanup and remediation matters most. Clearing tires, furniture, and debris removes the visible problem but can leave lead, arsenic and other elements in the soil, so a site may look restored while still carrying a measurable burden. EPA guidance is explicit that screening levels are not cleanup levels and that areas should be reinvestigated after contaminated soil is removed or treated to confirm that cleanup goals have been met (U.S. Environmental Protection Agency 1996b; U.S. Environmental Protection Agency, n.d.-b). For dumping sites, this means treating physical cleanup as a first step rather than the endpoint, and testing the soil at higher-risk sites, those with construction debris, chemicals, e-waste, burned material, or elevated screening results, before they are deemed safe for reuse. Depending on conditions, remediation may involve soil removal, capping, stabilization, phytoremediation, or restrictions on use (U.S. Environmental Protection Agency, n.d.-c).

The repository provides a practical structure for putting this into practice. By recording where dumping occurs, what materials are present, which sites show elevated Cf and Cdeg values, and where laboratory confirmation has been done, it lets a city triage sites and revisit them over time. Portable XRF makes the initial screening fast and affordable enough to apply across many sites, while more costly ICP-OES can be reserved for high-priority or remediation-bound locations. Used together within the repository, the two methods support defensible prioritization while keeping cost, turnaround, and logistical demands manageable for a resource-limited city.

### Comparison with other studies

The element assemblage reported here is broadly consistent with published work on soils at open and uncontrolled waste disposal sites, where lead, zinc and copper commonly dominate enrichment and arsenic and chromium appear at more modest levels (Ebong et al., 2019; Ihedioha et al., 2017; Vongdala et al., 2019). Studies of electronic waste handling similarly identify copper, lead and zinc as the characteristic signature (Houessionon et al., 2021), and epidemiological work at illegal dump and hazardous waste sites has associated proximity with adverse health outcomes at the population level (Fazzo et al., 2023). Our results differ from much of this literature in three respects. Most published assessments characterize a single disposal site in detail, whereas this study covers 45 sites distributed across one municipality, which is what allows the spatial unevenness and the influence of individual extreme sites to be seen. Most report surface soils only, whereas the paired-depth design here shows that enrichment is not confined to the surface. And most report indices without testing whether the resulting classification is robust to analytical method; our comparison of total and partial-extraction results shows that it is not.

Where directly comparable ranges are available our concentrations sit within those reported for informal dumping and electronic waste sites elsewhere. Adeyi and Oyeleke (2017) report 114 to 2840 mg/kg lead, 42.8 to 5390 mg/kg copper, 27.5 to 3420 mg/kg zinc, 11.0 to 128 mg/kg nickel and 94.0 to 325 mg/kg chromium in soils at e-waste dumpsites in Lagos and Ibadan. Our lead maximum of 2910 mg/kg is closely comparable, whereas our copper and zinc maxima of 1220 and 1630 mg/kg are several times lower, which is consistent with mixed municipal dumping being less enriched in those elements than sites dedicated to electronic waste processing. Our chromium range of 49.0 to 182 mg/kg is likewise narrower and lower, consistent with the lithogenic assignment of chromium in Sect. “Element associations and source groups” rather than a waste-derived source.

The systematic offset we observe between pXRF and acid-extraction results is also consistent with the intended use of field-portable XRF as a screening technique whose results are confirmed rather than replaced by laboratory analysis (U.S. Environmental Protection Agency, 2007). The strong correlations for lead, copper and zinc indicate that pXRF ranks sites reliably for the elements that matter most at these locations, while the weaker agreement for nickel and the variable agreement for manganese at depth mark the limits of that reliability.

### Strengths and limitations

This work has several strengths. It pairs field documentation with direct soil measurement, so the analysis rests on measured concentrations rather than complaints or visual inspection alone, and the site-level waste inventories let us connect element patterns to likely sources. The paired 0 to 2.5 cm and 15 cm sampling provides direct evidence on whether contamination persists at depth. The contamination factor and degree of contamination give accessible, action-oriented summaries of cumulative burden, and embedding portable XRF within a public repository makes the whole approach economical and repeatable for communities and municipalities. The agreement between XRF and ICP-OES for lead, copper, chromium, zinc, and arsenic further supports portable XRF as a reliable screening tool for this setting, with laboratory analysis reserved for confirmation.

Several limitations temper these strengths, and we state them explicitly. The number of sites varied across wards, and Ward 4 was represented by only two locations, so ward-level statistics for small wards are unstable. Because the distributions are strongly right-skewed, ward means are influenced disproportionately by one or two sites; this is most consequential in Ward 7, where the mean degree of contamination and the mean lead concentration both substantially exceed the corresponding medians, and we therefore report medians alongside means throughout. No certified reference material was analyzed by pXRF and no study-specific detection limits were established, so the pXRF results are screening-level; reproducibility was assessed instead by triplicate measurement and by comparing acquisition times, which showed poorer reproducibility for arsenic, copper and nickel than for lead, zinc and manganese. Weaker agreement between pXRF and ICP-OES for nickel, and variable agreement for manganese at depth, indicate that pXRF should complement rather than replace laboratory confirmation for those elements.

Several requested analyses were not performed and their absence bounds what can be concluded. Laboratory granulometry, total organic carbon and leaching or sequential extraction were not carried out, so no inference is drawn about element mobility or bioavailability. Soil series, texture, organic matter and carbon stock are survey-derived attributes at map-unit resolution rather than sample-specific determinations, so the absence of any correlation between organic matter and elemental burden should be read as inconclusive rather than as evidence against an organic matter association; determining total organic carbon on these samples is required to settle it. Chromium speciation was not determined, leaving the chromium hazard unresolved. Subsamples were composited without individual weighing, and because compositing removes within-site variance by construction, the within-site heterogeneity that the large standard deviations imply could not be quantified; retaining discrete subsamples at one or more sites would address this directly. No local population of uncontaminated soils was collected, which is the design that would permit the cumulative-distribution approach preferable to background-referenced indices. Photographs record site condition at the time of sampling but not soil profiles, since sampling composited discrete depth increments rather than exposing a profile face. Finally, the cross-sectional design cannot establish when dumping occurred, how long material had been present, or whether elements are actively migrating, and with 45 sites and presence-absence waste coding the study is not powered to explain why particular sites become hotspots.

## Conclusions

Addressing illegal dumping requires more than removing what can be seen. At 45 sites across Jackson, enrichment of potentially toxic elements extended to 15 cm at concentrations approaching those at the surface, with surface and subsurface concentrations significantly correlated for every element measured and nearly identical for lead (r = 0.982). Clearing surface waste, or even the uppermost soil, would therefore not remove the elemental burden. Lead, copper and zinc dominated enrichment, and lead exceeded the EPA residential soil screening level of 200 mg/kg at 11 of the 45 sites and the 600 mg/kg removal management level at 4, with a maximum of 2838 mg/kg.

Three further results bear on how such sites should be assessed. First, lithogenic and waste-derived contributions can be separated using field-portable screening alone: principal component analysis, hierarchical clustering and partial-extraction recovery ratios agree in grouping chromium and nickel with iron and separating lead, zinc, copper and arsenic from it. Second, the elements that dominate calculated health hazard, arsenic and manganese, are largely geogenic here, whereas the element driving regulatory exceedance is waste-derived; the child hazard index exceeded unity at 21 of 45 sites but at only one once arsenic was excluded, and adult hazard indices remained below unity everywhere. Third, index-based classification is method-dependent: contamination classes derived from total and from partial-extraction measurements agreed for only 1 of 13 paired sites. Contamination indices are therefore useful for ranking and prioritizing sites and unsuitable as measures of hazard, for which absolute regulatory thresholds are the appropriate reference.

Because the distributions are strongly right-skewed, ward-level means are driven by a small number of extreme sites, and the practical consequence is that remediation should be prioritized site by site rather than by ward. Environmental recovery should be defined by more than the absence of visible waste: it should include soil assessment, comparison against health-relevant regulatory values, and remediation where exceedances are found. Portable XRF screening embedded in a public repository, with laboratory confirmation reserved for high-priority sites, offers resource-limited cities a defensible and affordable route to that assessment, provided its limits are respected: it does not resolve speciation, it does not measure bioavailability, and its precision degrades for elements present at low concentrations. Future work should determine total organic carbon and particle size on these samples, add bioaccessibility and speciation measurements at the highest-burden sites, retain discrete subsamples to quantify within-site heterogeneity, and repeat sampling before and after cleanup so that geochemical evidence can be translated into exposure-prevention decisions. A site is not recovered when it merely looks clean; it is recovered when a city can show that the land is safer for the people who live, work, garden or play on it.

## Supplementary Information

Below is the link to the electronic supplementary material.

Supplementary file1 (DOCX 93 KB)

## Data Availability

The illegal dumping data repository generated in this study, including cataloged site locations, waste composition, and site-level contamination indices, is publicly available at https://communitynoiselab.org/illegaldumpingmap/. The underlying datasets generated and analysed during the current study are available from the corresponding author on reasonable request.

## Code availability

The custom code used for the analyses reported in this study is available from the corresponding author upon reasonable request.
