# Supplementary material for: Hidden toxicities: assessing city-wide contamination by potentially toxic elements from illegal dumping in Jackson, Mississippi
Source: Environ Geochem Health. 2026 Sep 24;48(15):603. doi: 10.1007/s10653-026-03450-y (PMC13612644; doi:10.1007/s10653-026-03450-y)
Supplement: Supplementary file 1 [file 10653_2026_3450_MOESM1_ESM.docx]

**Supplementary Material**

Hidden Toxicities: Assessing City-Wide Contamination by Potentially Toxic Elements from Illegal Dumping in Jackson, Mississippi

Walker, E. D.; Klevan, C.; Mathews, K.; Mandalapu, S. V.; Nica, C.; Ibrahim, N.

*Environmental Geochemistry and Health*

This file contains seven supplementary tables. Concentrations are reported in mg/kg to three significant figures. Sampling depths are given as 0 to 2.5 cm (surface) and 15 cm (subsurface). Contamination factors and the degree of contamination are calculated for the seven elements detected at every site and both depths; cadmium is excluded and reported descriptively, with an eight-element sensitivity analysis in Table S3.

**Table S1** Socioeconomic characterization of Jackson, Mississippi, by ward

|  | **Ward 1** | **Ward 2** | **Ward 3** | **Ward 4** | **Ward 5** | **Ward 6** | **Ward 7** |
| --- | --- | --- | --- | --- | --- | --- | --- |
| **Population and size** | | | | | | | |
| Population | 22,516 | 23,612 | 21,222 | 22,785 | 22,064 | 23,380 | 20,504 |
| Population under 5 years of age (%) | 12.54 | 12.43 | 11.17 | 14.51 | 13.34 | 13.85 | 9.69 |
| Under 18 years of age (%) | 24.80 | 22.04 | 22.98 | 25.70 | 20.03 | 27.49 | 18.84 |
| Over 65 years of age (%) | 14.24 | 20.07 | 18.20 | 14.65 | 12.89 | 12.62 | 15.45 |
| Non-Hispanic White (%) | 38.48 | 5.61 | 5.84 | 8.11 | 4.59 | 6.84 | 38.99 |
| Non-Hispanic Black or African American (%) | 55.99 | 90.84 | 90.21 | 88.78 | 92.19 | 86.66 | 55.77 |
| Non-Hispanic American Indian and Alaska Native (%) | 0.01 | 0.10 | 0.04 | 0.09 | 0.03 | 0.14 | 0.16 |
| Non-Hispanic Asian (%) | 0.73 | 0.12 | 0.00 | 0.07 | 0.26 | 0.16 | 0.42 |
| Hispanic or Latino (%) | 3.40 | 1.10 | 2.33 | 2.53 | 1.53 | 2.89 | 2.56 |
| **Language barriers** | | | | | | | |
| Limited English speaking (%) | 1.83 | 0.43 | 0.50 | 0.49 | 0.44 | 0.73 | 1.03 |
| **Employment and income** | | | | | | | |
| Unemployed civilians in the workforce (%) | 4.00 | 10.47 | 9.83 | 11.07 | 17.24 | 7.80 | 8.25 |
| Families with incomes less than twice the federal poverty level (%) | 23.96 | 48.38 | 66.22 | 55.66 | 67.10 | 52.10 | 47.59 |
| Renter-occupied housing units (%) | 32.59 | 45.57 | 56.46 | 44.90 | 68.79 | 38.38 | 58.53 |
| Median household income (USD) | 91037.41 | 42766.59 | 35566.00 | 45931.55 | 31819.39 | 56435.24 | 50477.35 |
| Population below the federal poverty level (%) | 11.48 | 23.11 | 33.98 | 23.92 | 39.27 | 26.11 | 27.95 |
| **Health and well-being** | | | | | | | |
| Low life expectancy (%) | 0.16 | 0.22 | 0.25 | 0.24 | 0.25 | 0.26 | 0.23 |
| **Summary measures** | | | | | | | |
| EJ Demographic Index (mean of low-income and people-of-color percentages) | 1.47 | 2.59 | 2.99 | 2.73 | 3.07 | 2.66 | 2.02 |

*Source: U.S. Census Bureau (2022), American Community Survey 5-year estimates (2018–2022). These variables describe the study area and are not analysed in this study.*

**Table S2** Descriptive statistics of soil concentrations of potentially toxic elements (mg/kg) by ward and depth

| **Ward** | **Element** | **Depth (cm)** | **n** | **Mean** | **SD** | **Median** | **P25** | **P75** | **Min** | **Max** | **EPA resident soil (mg/kg)** |
| --- | --- | --- | --- | --- | --- | --- | --- | --- | --- | --- | --- |
| 2 | Cr | 0 to 2.5 | 8 | 103 | 34 | 102 | 91.2 | 106 | 49 | 173 | 1.2 × 10⁵ |
| 2 | Cr | 15 | 8 | 103 | 29 | 102 | 86.5 | 116 | 63.5 | 155 | 1.2 × 10⁵ |
| 2 | Mn | 0 to 2.5 | 8 | 756 | 227 | 670 | 615 | 892 | 470 | 1130 | 1,800 |
| 2 | Mn | 15 | 8 | 994 | 1090 | 623 | 576 | 710 | 356 | 3680 | 1,800 |
| 2 | Ni | 0 to 2.5 | 8 | 27.4 | 11 | 23.8 | 21.2 | 29.2 | 17.5 | 51.3 | 1,400 |
| 2 | Ni | 15 | 8 | 30.3 | 11 | 27.2 | 23.7 | 32.8 | 20 | 55 | 1,400 |
| 2 | Cu | 0 to 2.5 | 8 | 92.6 | 135 | 32.6 | 16 | 105 | 11.3 | 410 | 3,100 |
| 2 | Cu | 15 | 8 | 87.1 | 102 | 57.3 | 15.7 | 101 | 13 | 316 | 3,100 |
| 2 | Zn | 0 to 2.5 | 8 | 368 | 520 | 154 | 119 | 333 | 85.3 | 1630 | 23,000 |
| 2 | Zn | 15 | 8 | 296 | 258 | 235 | 97.3 | 370 | 81.7 | 835 | 23,000 |
| 2 | As | 0 to 2.5 | 8 | 13.9 | 7.4 | 11.8 | 10.7 | 14.2 | 6.5 | 31 | 0.68 |
| 2 | As | 15 | 8 | 14.1 | 5.05 | 13 | 11.9 | 14.7 | 8 | 25.3 | 0.68 |
| 2 | Pb | 0 to 2.5 | 8 | 135 | 228 | 47.2 | 26 | 86 | 25 | 690 | 200 |
| 2 | Pb | 15 | 8 | 104 | 133 | 44.2 | 26.7 | 101 | 17.7 | 396 | 200 |
| 3 | Cr | 0 to 2.5 | 6 | 100 | 21.4 | 102 | 84.5 | 115 | 72.5 | 128 | 1.2 × 10⁵ |
| 3 | Cr | 15 | 6 | 107 | 23.2 | 109 | 87.9 | 125 | 78.5 | 134 | 1.2 × 10⁵ |
| 3 | Mn | 0 to 2.5 | 6 | 669 | 254 | 603 | 507 | 798 | 390 | 1080 | 1,800 |
| 3 | Mn | 15 | 6 | 724 | 331 | 612 | 507 | 826 | 436 | 1320 | 1,800 |
| 3 | Ni | 0 to 2.5 | 6 | 27.4 | 8.03 | 27.2 | 21.4 | 30.1 | 19 | 40.7 | 1,400 |
| 3 | Ni | 15 | 6 | 26.9 | 7.98 | 26.3 | 23.1 | 29.8 | 16 | 39.7 | 1,400 |
| 3 | Cu | 0 to 2.5 | 6 | 44.8 | 31.1 | 34.8 | 20.9 | 68.5 | 15 | 88.3 | 3,100 |
| 3 | Cu | 15 | 6 | 55.4 | 53.8 | 33.2 | 20.6 | 62.5 | 19 | 158 | 3,100 |
| 3 | Zn | 0 to 2.5 | 6 | 245 | 123 | 260 | 136 | 344 | 101 | 383 | 23,000 |
| 3 | Zn | 15 | 6 | 236 | 121 | 248 | 133 | 335 | 91 | 371 | 23,000 |
| 3 | As | 0 to 2.5 | 6 | 14.8 | 4.46 | 13.1 | 12.1 | 16.5 | 10.5 | 22.5 | 0.68 |
| 3 | As | 15 | 6 | 15.9 | 6.63 | 13.1 | 11.1 | 19.7 | 10.5 | 26.7 | 0.68 |
| 3 | Pb | 0 to 2.5 | 6 | 98.4 | 63.5 | 84 | 57.2 | 124 | 32 | 205 | 200 |
| 3 | Pb | 15 | 6 | 111 | 83.8 | 95 | 57.1 | 127 | 29 | 261 | 200 |
| 4 | Cr | 0 to 2.5 | 2 | 93.5 | 3.54 | 93.5 | 92.2 | 94.7 | 91 | 96 | 1.2 × 10⁵ |
| 4 | Cr | 15 | 2 | 75 | 8.49 | 75 | 72 | 78 | 69 | 81 | 1.2 × 10⁵ |
| 4 | Mn | 0 to 2.5 | 2 | 805 | 244 | 805 | 719 | 892 | 632 | 978 | 1,800 |
| 4 | Mn | 15 | 2 | 798 | 220 | 798 | 720 | 875 | 642 | 953 | 1,800 |
| 4 | Ni | 0 to 2.5 | 2 | 22 | 8.49 | 22 | 19 | 25 | 16 | 28 | 1,400 |
| 4 | Ni | 15 | 2 | 22.8 | 8.72 | 22.8 | 19.8 | 25.9 | 16.7 | 29 | 1,400 |
| 4 | Cu | 0 to 2.5 | 2 | 41.5 | 19.1 | 41.5 | 34.8 | 48.2 | 28 | 55 | 3,100 |
| 4 | Cu | 15 | 2 | 62.2 | 64.3 | 62.2 | 39.4 | 84.9 | 16.7 | 108 | 3,100 |
| 4 | Zn | 0 to 2.5 | 2 | 303 | 179 | 303 | 240 | 366 | 176 | 429 | 23,000 |
| 4 | Zn | 15 | 2 | 453 | 429 | 453 | 301 | 605 | 150 | 756 | 23,000 |
| 4 | As | 0 to 2.5 | 2 | 13.7 | 5.19 | 13.7 | 11.8 | 15.5 | 10 | 17.3 | 0.68 |
| 4 | As | 15 | 2 | 11.7 | 2.83 | 11.7 | 10.7 | 12.7 | 9.67 | 13.7 | 0.68 |
| 4 | Pb | 0 to 2.5 | 2 | 292 | 281 | 292 | 193 | 391 | 93.5 | 490 | 200 |
| 4 | Pb | 15 | 2 | 420 | 425 | 420 | 269 | 570 | 119 | 721 | 200 |
| 5 | Cr | 0 to 2.5 | 15 | 94.5 | 23.4 | 90 | 86.6 | 104 | 57.5 | 148 | 1.2 × 10⁵ |
| 5 | Cr | 15 | 15 | 102 | 29.2 | 105 | 88.2 | 111 | 53 | 182 | 1.2 × 10⁵ |
| 5 | Mn | 0 to 2.5 | 15 | 499 | 140 | 474 | 402 | 531 | 356 | 908 | 1,800 |
| 5 | Mn | 15 | 15 | 511 | 111 | 501 | 437 | 556 | 331 | 750 | 1,800 |
| 5 | Ni | 0 to 2.5 | 15 | 22.4 | 4.06 | 22.3 | 20.8 | 24.5 | 14.5 | 30 | 1,400 |
| 5 | Ni | 15 | 15 | 23.2 | 3.69 | 23.3 | 21.8 | 25.2 | 14.3 | 30 | 1,400 |
| 5 | Cu | 0 to 2.5 | 15 | 74.5 | 78 | 52 | 20.2 | 77.5 | 13.3 | 268 | 3,100 |
| 5 | Cu | 15 | 15 | 59.2 | 55.9 | 37.5 | 18.8 | 90.8 | 11.3 | 174 | 3,100 |
| 5 | Zn | 0 to 2.5 | 15 | 351 | 213 | 394 | 191 | 481 | 60 | 711 | 23,000 |
| 5 | Zn | 15 | 15 | 292 | 200 | 203 | 139 | 397 | 62 | 731 | 23,000 |
| 5 | As | 0 to 2.5 | 15 | 14.6 | 5.2 | 12.5 | 10.9 | 17.7 | 8 | 27 | 0.68 |
| 5 | As | 15 | 15 | 15.2 | 5.44 | 13 | 12 | 16.6 | 10 | 28 | 0.68 |
| 5 | Pb | 0 to 2.5 | 15 | 218 | 216 | 138 | 61.4 | 286 | 13 | 663 | 200 |
| 5 | Pb | 15 | 15 | 195 | 189 | 116 | 52.2 | 278 | 22 | 587 | 200 |
| 6 | Cr | 0 to 2.5 | 7 | 93.9 | 14.7 | 101 | 87.8 | 104 | 67.3 | 106 | 1.2 × 10⁵ |
| 6 | Cr | 15 | 7 | 107 | 15 | 109 | 98.7 | 114 | 82.7 | 128 | 1.2 × 10⁵ |
| 6 | Mn | 0 to 2.5 | 7 | 517 | 175 | 469 | 401 | 649 | 287 | 764 | 1,800 |
| 6 | Mn | 15 | 7 | 426 | 175 | 410 | 312 | 562 | 175 | 654 | 1,800 |
| 6 | Ni | 0 to 2.5 | 7 | 24.6 | 7.34 | 24.3 | 20.8 | 26.8 | 14.7 | 38 | 1,400 |
| 6 | Ni | 15 | 7 | 25.7 | 10.6 | 22 | 19.8 | 27.5 | 15.7 | 47.7 | 1,400 |
| 6 | Cu | 0 to 2.5 | 7 | 66.3 | 73.4 | 34.5 | 22.5 | 86.1 | 7.67 | 205 | 3,100 |
| 6 | Cu | 15 | 7 | 52 | 66.9 | 20 | 15.8 | 55.3 | 7.67 | 194 | 3,100 |
| 6 | Zn | 0 to 2.5 | 7 | 196 | 106 | 184 | 114 | 251 | 79.7 | 376 | 23,000 |
| 6 | Zn | 15 | 7 | 178 | 101 | 151 | 93.7 | 264 | 66 | 314 | 23,000 |
| 6 | As | 0 to 2.5 | 7 | 15.4 | 6.44 | 13.5 | 11.9 | 18.9 | 7.67 | 25 | 0.68 |
| 6 | As | 15 | 7 | 14.6 | 5.11 | 14 | 11.3 | 17 | 8.33 | 23 | 0.68 |
| 6 | Pb | 0 to 2.5 | 7 | 60 | 21.1 | 53.3 | 50.8 | 70.3 | 30.7 | 94 | 200 |
| 6 | Pb | 15 | 7 | 61.6 | 25.4 | 63.3 | 44.9 | 73.7 | 27.7 | 103 | 200 |
| 7 | Cr | 0 to 2.5 | 7 | 97.5 | 14.2 | 103 | 88.2 | 108 | 75 | 113 | 1.2 × 10⁵ |
| 7 | Cr | 15 | 7 | 107 | 18.8 | 105 | 95.7 | 125 | 79.5 | 126 | 1.2 × 10⁵ |
| 7 | Mn | 0 to 2.5 | 7 | 971 | 334 | 969 | 779 | 1140 | 506 | 1490 | 1,800 |
| 7 | Mn | 15 | 7 | 1000 | 301 | 999 | 792 | 1170 | 624 | 1470 | 1,800 |
| 7 | Ni | 0 to 2.5 | 7 | 24.7 | 2.53 | 24 | 23 | 26.3 | 21.7 | 28.3 | 1,400 |
| 7 | Ni | 15 | 7 | 25.2 | 3.7 | 26.8 | 24.7 | 27.3 | 17.5 | 28.3 | 1,400 |
| 7 | Cu | 0 to 2.5 | 7 | 261 | 428 | 96.3 | 72.8 | 158 | 47 | 1220 | 3,100 |
| 7 | Cu | 15 | 7 | 149 | 121 | 124 | 77.2 | 156 | 49 | 406 | 3,100 |
| 7 | Zn | 0 to 2.5 | 7 | 474 | 262 | 495 | 286 | 552 | 183 | 962 | 23,000 |
| 7 | Zn | 15 | 7 | 486 | 310 | 352 | 288 | 606 | 190 | 1070 | 23,000 |
| 7 | As | 0 to 2.5 | 7 | 17.6 | 12.8 | 11.3 | 10.7 | 17.8 | 10.3 | 44.5 | 0.68 |
| 7 | As | 15 | 7 | 20 | 15.2 | 13.7 | 12.4 | 19.1 | 10 | 53 | 0.68 |
| 7 | Pb | 0 to 2.5 | 7 | 602 | 984 | 92.7 | 75.7 | 577 | 53 | 2770 | 200 |
| 7 | Pb | 15 | 7 | 599 | 1040 | 97.3 | 85.5 | 477 | 57.7 | 2910 | 200 |

*All seven elements were detected at every site at both depths. Cadmium is excluded; it was above the reporting threshold at one of 45 sites at the surface (18 mg/kg) and three of 45 sites at 15 cm (19, 24 and 27 mg/kg). The final column gives the current US EPA residential soil screening level for each element (U.S. Environmental Protection Agency 2026); the level for chromium is that for trivalent chromium.*

**Table S3** Descriptive statistics of the contamination factor (Cf) and degree of contamination (Cdeg) by ward

| **Ward** | **Index** | **n** | **Mean** | **SD** | **Median** | **P25** | **P75** | **Min** | **Max** |
| --- | --- | --- | --- | --- | --- | --- | --- | --- | --- |
| 2 | Cf Cr | 8 | 2.75 | 0.8 | 2.6 | 2.52 | 2.89 | 1.5 | 4.36 |
| 2 | Cf Mn | 8 | 1.72 | 1.25 | 1.24 | 1.2 | 1.66 | 0.811 | 4.73 |
| 2 | Cf Ni | 8 | 0.943 | 0.246 | 0.835 | 0.771 | 1.12 | 0.697 | 1.37 |
| 2 | Cf Cu | 8 | 6.29 | 8.2 | 3.82 | 1.15 | 6.06 | 0.851 | 25.4 |
| 2 | Cf Zn | 8 | 8.54 | 9.7 | 6.07 | 2.76 | 8.13 | 2.22 | 31.7 |
| 2 | Cf As | 8 | 2.03 | 0.898 | 1.85 | 1.65 | 2.07 | 1.05 | 4.08 |
| 2 | Cf Pb | 8 | 4.54 | 6.69 | 1.86 | 0.998 | 4.29 | 0.83 | 20.6 |
| 2 | Cdeg (7 elements) | 8 | 26.8 | 25.1 | 21.2 | 11.9 | 25.1 | 10.2 | 86.6 |
| 2 | Cdeg (8 elements, sensitivity) | 8 | 28.3 | 24.8 | 24.1 | 11.9 | 28.5 | 10.2 | 86.6 |
| 3 | Cf Cr | 6 | 2.77 | 0.538 | 2.97 | 2.32 | 3.05 | 2.09 | 3.4 |
| 3 | Cf Mn | 6 | 1.37 | 0.568 | 1.17 | 0.946 | 1.59 | 0.905 | 2.36 |
| 3 | Cf Ni | 6 | 0.888 | 0.258 | 0.876 | 0.723 | 0.977 | 0.58 | 1.31 |
| 3 | Cf Cu | 6 | 3.5 | 2.8 | 2.38 | 1.45 | 4.9 | 1.19 | 8.18 |
| 3 | Cf Zn | 6 | 6.19 | 3.14 | 6.53 | 3.46 | 8.73 | 2.47 | 9.7 |
| 3 | Cf As | 6 | 2.23 | 0.765 | 1.9 | 1.68 | 2.9 | 1.52 | 3.2 |
| 3 | Cf Pb | 6 | 3.97 | 2.79 | 3.43 | 2.18 | 4.75 | 1.16 | 8.87 |
| 3 | Cdeg (7 elements) | 6 | 20.9 | 9.53 | 19.8 | 13.2 | 26.5 | 11.3 | 34.7 |
| 3 | Cdeg (8 elements, sensitivity) | 6 | 20.9 | 9.53 | 19.8 | 13.2 | 26.5 | 11.3 | 34.7 |
| 4 | Cf Cr | 2 | 2.25 | 0.066 | 2.25 | 2.22 | 2.27 | 2.2 | 2.29 |
| 4 | Cf Mn | 2 | 1.57 | 0.457 | 1.57 | 1.41 | 1.74 | 1.25 | 1.9 |
| 4 | Cf Ni | 2 | 0.733 | 0.281 | 0.733 | 0.633 | 0.832 | 0.534 | 0.931 |
| 4 | Cf Cu | 2 | 3.62 | 2.92 | 3.62 | 2.59 | 4.66 | 1.56 | 5.69 |
| 4 | Cf Zn | 2 | 9.71 | 7.81 | 9.71 | 6.95 | 12.5 | 4.19 | 15.2 |
| 4 | Cf As | 2 | 1.84 | 0.581 | 1.84 | 1.63 | 2.04 | 1.43 | 2.25 |
| 4 | Cf Pb | 2 | 13.5 | 13.4 | 13.5 | 8.79 | 18.3 | 4.04 | 23 |
| 4 | Cdeg (7 elements) | 2 | 33.3 | 24.6 | 33.3 | 24.6 | 42 | 15.9 | 50.7 |
| 4 | Cdeg (8 elements, sensitivity) | 2 | 33.3 | 24.6 | 33.3 | 24.6 | 42 | 15.9 | 50.7 |
| 5 | Cf Cr | 15 | 2.62 | 0.653 | 2.69 | 2.35 | 2.9 | 1.56 | 4.05 |
| 5 | Cf Mn | 15 | 0.992 | 0.203 | 0.963 | 0.876 | 1.07 | 0.713 | 1.5 |
| 5 | Cf Ni | 15 | 0.746 | 0.121 | 0.746 | 0.723 | 0.769 | 0.471 | 0.98 |
| 5 | Cf Cu | 15 | 4.68 | 4.17 | 3.27 | 1.28 | 7.33 | 0.874 | 13.4 |
| 5 | Cf Zn | 15 | 8.27 | 4.95 | 9.76 | 4.55 | 10.8 | 1.57 | 18.1 |
| 5 | Cf As | 15 | 2.16 | 0.73 | 1.98 | 1.7 | 2.44 | 1.3 | 3.99 |
| 5 | Cf Pb | 15 | 7.85 | 7.66 | 4.58 | 2.16 | 10.7 | 0.665 | 23.8 |
| 5 | Cdeg (7 elements) | 15 | 27.3 | 15.1 | 25.8 | 16.2 | 34.9 | 8.42 | 60.5 |
| 5 | Cdeg (8 elements, sensitivity) | 15 | 28.6 | 15.6 | 28.5 | 16.2 | 38.3 | 8.42 | 60.5 |
| 6 | Cf Cr | 7 | 2.67 | 0.381 | 2.76 | 2.52 | 2.9 | 2 | 3.1 |
| 6 | Cf Mn | 7 | 0.927 | 0.343 | 0.863 | 0.7 | 1.19 | 0.454 | 1.39 |
| 6 | Cf Ni | 7 | 0.822 | 0.29 | 0.752 | 0.647 | 0.886 | 0.534 | 1.4 |
| 6 | Cf Cu | 7 | 4.14 | 4.87 | 1.91 | 1.34 | 4.94 | 0.536 | 14 |
| 6 | Cf Zn | 7 | 4.81 | 2.56 | 5.19 | 2.67 | 6.19 | 1.87 | 8.87 |
| 6 | Cf As | 7 | 2.17 | 0.826 | 1.99 | 1.67 | 2.57 | 1.26 | 3.48 |
| 6 | Cf Pb | 7 | 2.31 | 0.875 | 2.22 | 1.82 | 2.74 | 1.11 | 3.75 |
| 6 | Cdeg (7 elements) | 7 | 17.8 | 8.38 | 15.6 | 12 | 24 | 8.05 | 29.3 |
| 6 | Cdeg (8 elements, sensitivity) | 7 | 17.8 | 8.38 | 15.6 | 12 | 24 | 8.05 | 29.3 |
| 7 | Cf Cr | 7 | 2.73 | 0.411 | 2.79 | 2.39 | 3.1 | 2.18 | 3.16 |
| 7 | Cf Mn | 7 | 1.94 | 0.616 | 1.89 | 1.51 | 2.27 | 1.22 | 2.9 |
| 7 | Cf Ni | 7 | 0.815 | 0.0918 | 0.828 | 0.776 | 0.88 | 0.651 | 0.915 |
| 7 | Cf Cu | 7 | 14.3 | 19.1 | 7.69 | 5.24 | 11 | 3.36 | 56.9 |
| 7 | Cf Zn | 7 | 12.3 | 7.17 | 12.1 | 7.39 | 14.3 | 4.79 | 26.1 |
| 7 | Cf As | 7 | 2.72 | 2.02 | 1.74 | 1.71 | 2.64 | 1.55 | 7.07 |
| 7 | Cf Pb | 7 | 22.8 | 38.5 | 3.53 | 3.11 | 20 | 2.1 | 108 |
| 7 | Cdeg (7 elements) | 7 | 57.7 | 48.3 | 33.5 | 26.4 | 70.6 | 23.5 | 153 |
| 7 | Cdeg (8 elements, sensitivity) | 7 | 59.7 | 52.8 | 33.5 | 26.4 | 70.6 | 23.5 | 167 |

*Cdeg (7 elements) is the index reported in the main text. Cdeg (8 elements) includes cadmium with non-detects assigned zero and is shown as a sensitivity analysis: it changes the classification of two sites and leaves the ward ranking unaltered.*

**Table S4a** Site identification, location and soil properties for all 45 sites

| **Site ID** | **Ward** | **Latitude** | **Longitude** | **Perimeter (m)** | **Soil pH** | **Texture 0 to 2.5 cm** | **Texture 15 cm** | **Soil series** |
| --- | --- | --- | --- | --- | --- | --- | --- | --- |
| 01_06042024_MinervaSt | 5 | 32.30083 | -90.19667 | 88 | 3.2 | silt loam | silt loam | Loring-Urban |
| 02_06122024_HartfieldSt | 7 | 32.3385 | -90.18302 | 102 | 5.6 | silt loam | silty clay loam | Byram-Urban |
| 03_06242024_ClaiborneAve | 2 | 32.3082 | -90.22278 | 128 | 8 | silt loam | silt loam | Loring-Urban |
| 04_07162024_WCapitolSt_Site1 | 5 | 32.31108 | -90.20701 | 108 | 6.1 | silt loam | silt loam | Loring-Urban |
| 05_07302024_ByrdDr | 4 | 32.32535 | -90.24419 | 250 | 8.7 | silt loam | silt loam | Adler |
| 06_08302024_WCapitolSt_Site2 | 5 | 32.30643 | -90.20028 | 168 | 6.8 | silt loam | silt loam | Loring-Urban |
| 07_08122024_GlenErinSt | 6 | 32.26297 | -90.23679 | 250 | 7 | silt loam | silty clay loam | Siwell-Urban |
| 08_08122024_MeadowLn | 6 | 32.25676 | -90.23513 | 100 | 7 | silt loam | silty clay loam | Byram-Urban |
| 09_11142024_MetroCenter | 5 | 32.30208 | -90.25164 | 265 | 5.9 | silt | silty clay loam | Siwell |
| 10_11142024_TeresaDr | 6 | 32.26735 | -90.24191 | 191 | 7 | silt loam | silty clay loam | Siwell-Urban |
| 11_11142024_RevereSt | 6 | 32.26663 | -90.24078 | 130 | 4.3 | silt loam | silty clay loam | Siwell-Urban |
| 12_11142024_CooperRd | 6 | 32.2534 | -90.25569 | 288 | 3.1 | silt loam | silt loam | Calloway-Urban |
| 13_11142024_ForestHillRd | 6 | 32.24823 | -90.26643 | 424 | 3 | silt loam | silt loam | Loring-Urban |
| 14_11142024_MccluerRd | 6 | 32.23969 | -90.27778 | 88.1 | 4.3 | silt loam | silty clay loam | Byram-Urban |
| 15_11142024_RobinsonRd | 5 | 32.29528 | -90.25912 | 100 | 4.3 | silt loam | silt loam | Loring-Urban |
| 16_11142024_BarnettDr | 5 | 32.3029 | -90.24755 | 343 | 6.3 | silt loam | silty clay loam | Byram |
| 17_11142024_NPrentissSt | 5 | 32.31765 | -90.20836 | 111 | 5.3 | silt loam | silt loam | Riedtown |
| 18_11142024_LongSt | 5 | 32.3171 | -90.21032 | 260 | 5 | silt loam | silt loam | Loring-Urban |
| 19_11142024_BonAirSt | 5 | 32.31598 | -90.21533 | 147 | 5.7 | silt loam | silty clay loam | Siwell-Urban |
| 20_11142024_CollierAve | 3 | 32.35025 | -90.19218 | 50 | 3 | silt loam | silt loam | Riedtown |
| 22_11142024_MartinLutherKingJrDr | 5 | 32.31179 | -90.19533 | 66 | 3.7 | clay loam | clay loam | Urban Land |
| 23_11142024_JohnRLynchSt | 7 | 32.29769 | -90.19377 | 60.4 | 6.5 | silt loam | silt loam | Loring-Urban |
| 24_11142024_CliftonSt | 7 | 32.29648 | -90.19442 | 112 | 8.3 | silt loam | silt loam | Loring-Urban |
| 25_11142024_HookerSt | 7 | 32.29427 | -90.1968 | 286 | 3.9 | silt loam | silt loam | Riedtown |
| 26_11142024_WCountyLineRd_Site1 | 2 | 32.39859 | -90.15112 | 365 | 3.7 | silt loam | silt loam | Riedtown |
| 27_11142024_BrownSt | 2 | 32.39306 | -90.15905 | 384 | 5.1 | silt loam | silt loam | Byram-Urban |
| 28_11142024_GilbertAve | 2 | 32.37347 | -90.24854 | 222 | 4.3 | silt loam | silt loam | Loring-Urban |
| 29_11142024_Highway49_Site1 | 2 | 32.39516 | -90.27193 | 179 | 5.5 | silt loam | silt loam | Oaklimeter |
| 32_11142024_WinterSt | 5 | 32.29024 | -90.20401 | 175 | 8.1 | silt loam | silty clay loam | Byram |
| 33_11142024_ClearySt | 5 | 32.29826 | -90.20218 | 70 | 8.1 | silt loam | silt loam | Loring-Urban |
| 34_11142024_MedgarEversBlvd_Site1 | 3 | 32.33362 | -90.20626 | 117 | 6.6 | silt loam | silt loam | Loring-Urban |
| 35_11142024_MedgarEversBlvd_Site2 | 3 | 32.32964 | -90.20102 | 267 | 5.2 | silt loam | silt loam | Riedtown |
| 36_02212025_JohnsonSt | 3 | 32.3431 | -90.19374 | 17.3 | 4.3 | silt loam | silt loam | Loring-Urban |
| 39_02212025_WMcDowellRd_Site1 | 7 | 32.26842 | -90.2386 | 48.4 | 6.9 | silt loam | silt loam | Calloway-Urban |
| 42_02212025_FlorenceAve | 5 | 32.29367 | -90.20704 | 35 | 6.8 | silt loam | silt loam | Loring-Urban |
| 49_02212025_SunrayDr | 4 | 32.35432 | -90.22353 | 139 | 6.5 | silt loam | silt loam | Loring-Urban |
| 50_02212025_PercyVSimpsonDr | 3 | 32.3529 | -90.22627 | 372 | 5 | silt loam | silt loam | Byram-Urban |
| 51_02212025_KingswoodAve | 7 | 32.26587 | -90.21091 | 170 | 6 | silt loam | silty clay loam | Siwell-Urban |
| 52_02212025_LaSalleSt | 7 | 32.2642 | -90.21096 | 431 | 6.8 | silt loam | silty clay loam | Siwell-Urban |
| 53_02212025_RoseSt | 5 | 32.29952 | -90.20158 | 86 | 5.2 | silt loam | silt loam | Loring-Urban |
| 55_06252025_WCountyLineRd_Site2 | 2 | 32.40003 | -90.25297 | 74 | 6 | silt loam | silt loam | Riedtown |
| 56_06252025_BrownSt_Site 2 | 2 | 32.39842 | -90.15878 | 66.7 | 7.1 | silt loam | silty clay loam | Byram-Urban |
| 57_06302025_StateSt_Site1 | 3 | 32.36153 | -90.17221 | 38.7 | 6.9 | silt loam | silt loam | Loring-Urban |
| 61_06242025_ShirleyAve_Site1 | 5 | 32.29455 | -90.2092 | 50.5 | 6.6 | silt loam | silt loam | Loring-Urban |
| 62_06252025_HildaDr_Site1 | 2 | 32.39997 | -90.24701 | 1260 | 6.9 | silt loam | silt loam | Loring-Memphis |

*Soil pH was measured in the field with a probe. Texture and soil series are survey-derived attributes obtained by spatial join to the USDA Natural Resources Conservation Service soil survey and were not determined on the collected samples.*

**Table S4b** Waste categories recorded at each site

| **Site ID** | **Ward** | **Tires** | **Furniture** | **Domestic** | **Construction** | **Chemicals** | **E-waste** | **Yard waste** |
| --- | --- | --- | --- | --- | --- | --- | --- | --- |
| 01_06042024_MinervaSt | 5 | 1 | 1 | 1 | 1 | 0 | 0 | 0 |
| 02_06122024_HartfieldSt | 7 | 1 | 1 | 1 | 1 | 1 | 1 | 1 |
| 03_06242024_ClaiborneAve | 2 | 1 | 1 | 1 | 1 | 0 | 0 | 0 |
| 04_07162024_WCapitolSt_Site1 | 5 | 1 | 0 | 1 | 1 | 1 | 0 | 1 |
| 05_07302024_ByrdDr | 4 | 1 | 1 | 1 | 1 | 1 | 1 | 0 |
| 06_08302024_WCapitolSt_Site2 | 5 | 1 | 0 | 1 | 1 | 0 | 0 | 0 |
| 07_08122024_GlenErinSt | 6 | 1 | 1 | 0 | 1 | 0 | 0 | 0 |
| 08_08122024_MeadowLn | 6 | 1 | 0 | 1 | 1 | 0 | 0 | 0 |
| 09_11142024_MetroCenter | 5 | 1 | 1 | 1 | 1 | 1 | 1 | 1 |
| 10_11142024_TeresaDr | 6 | 1 | 0 | 1 | 1 | 1 | 0 | 0 |
| 11_11142024_RevereSt | 6 | 1 | 1 | 1 | 1 | 1 | 0 | 0 |
| 12_11142024_CooperRd | 6 | 1 | 1 | 1 | 1 | 1 | 1 | 0 |
| 13_11142024_ForestHillRd | 6 | 1 | 0 | 1 | 1 | 1 | 0 | 0 |
| 14_11142024_MccluerRd | 6 | 1 | 1 | 1 | 1 | 1 | 1 | 0 |
| 15_11142024_RobinsonRd | 5 | 1 | 1 | 1 | 1 | 0 | 0 | 0 |
| 16_11142024_BarnettDr | 5 | 1 | 1 | 1 | 1 | 1 | 1 | 1 |
| 17_11142024_NPrentissSt | 5 | 1 | 1 | 1 | 0 | 1 | 0 | 0 |
| 18_11142024_LongSt | 5 | 1 | 1 | 1 | 0 | 1 | 0 | 0 |
| 19_11142024_BonAirSt | 5 | 1 | 1 | 1 | 0 | 1 | 1 | 0 |
| 20_11142024_CollierAve | 3 | 1 | 0 | 1 | 0 | 0 | 0 | 0 |
| 22_11142024_MartinLutherKingJrDr | 5 | 1 | 1 | 1 | 0 | 0 | 0 | 0 |
| 23_11142024_JohnRLynchSt | 7 | 1 | 1 | 1 | 0 | 0 | 1 | 0 |
| 24_11142024_CliftonSt | 7 | 1 | 1 | 1 | 1 | 0 | 0 | 0 |
| 25_11142024_HookerSt | 7 | 1 | 1 | 1 | 1 | 0 | 0 | 0 |
| 26_11142024_WCountyLineRd_Site1 | 2 | 0 | 0 | 1 | 1 | 0 | 1 | 0 |
| 27_11142024_BrownSt | 2 | 1 | 1 | 1 | 0 | 1 | 0 | 0 |
| 28_11142024_GilbertAve | 2 | 1 | 1 | 1 | 1 | 0 | 0 | 0 |
| 29_11142024_Highway49_Site1 | 2 | 1 | 1 | 1 | 1 | 0 | 0 | 0 |
| 32_11142024_WinterSt | 5 | 0 | 1 | 1 | 1 | 1 | 0 | 0 |
| 33_11142024_ClearySt | 5 | 1 | 1 | 0 | 1 | 0 | 1 | 0 |
| 34_11142024_MedgarEversBlvd_Site1 | 3 | 1 | 1 | 1 | 1 | 1 | 1 | 0 |
| 35_11142024_MedgarEversBlvd_Site2 | 3 | 1 | 0 | 0 | 1 | 1 | 1 | 0 |
| 36_02212025_JohnsonSt | 3 | 1 | 0 | 1 | 0 | 0 | 0 | 0 |
| 39_02212025_WMcDowellRd_Site1 | 7 | 1 | 1 | 1 | 1 | 1 | 0 | 1 |
| 42_02212025_FlorenceAve | 5 | 1 | 0 | 1 | 1 | 1 | 0 | 0 |
| 49_02212025_SunrayDr | 4 | 0 | 0 | 1 | 1 | 0 | 0 | 0 |
| 50_02212025_PercyVSimpsonDr | 3 | 1 | 1 | 1 | 0 | 0 | 0 | 0 |
| 51_02212025_KingswoodAve | 7 | 1 | 1 | 1 | 0 | 0 | 0 | 1 |
| 52_02212025_LaSalleSt | 7 | 0 | 1 | 0 | 1 | 1 | 1 | 0 |
| 53_02212025_RoseSt | 5 | 0 | 0 | 1 | 0 | 0 | 0 | 0 |
| 55_06252025_WCountyLineRd_Site2 | 2 | 0 | 0 | 1 | 1 | 0 | 0 | 0 |
| 56_06252025_BrownSt_Site 2 | 2 | 1 | 1 | 1 | 1 | 0 | 0 | 0 |
| 57_06302025_StateSt_Site1 | 3 | 0 | 1 | 1 | 1 | 0 | 0 | 0 |
| 61_06242025_ShirleyAve_Site1 | 5 | 1 | 1 | 1 | 0 | 1 | 1 | 0 |
| 62_06252025_HildaDr_Site1 | 2 | 1 | 0 | 1 | 1 | 0 | 0 | 0 |

*1 denotes the category was recorded as present, 0 as absent. Categories were not quantified by volume or mass. No waste was removed from any site by the study team.*

**Table S4c** Element concentrations at 0 to 2.5 cm (mg/kg) for all 45 sites

| **Site ID** | **Ward** | **Cr** | **Mn** | **Ni** | **Cu** | **Zn** | **As** | **Pb** | **Fe** | **Cd** |
| --- | --- | --- | --- | --- | --- | --- | --- | --- | --- | --- |
| 01_06042024_MinervaSt | 5 | 85.7 | 531 | 21.3 | 209 | 680 | 27 | 543 | 29500 | n.d. |
| 02_06122024_HartfieldSt | 7 | 105 | 1490 | 25 | 47 | 321 | 11 | 92.7 | 20400 | n.d. |
| 03_06242024_ClaiborneAve | 2 | 108 | 1030 | 22.7 | 410 | 1630 | 31 | 690 | 18400 | n.d. |
| 04_07162024_WCapitolSt_Site1 | 5 | 88.5 | 448 | 22.5 | 56 | 459 | 19 | 590 | 19600 | n.d. |
| 05_07302024_ByrdDr | 4 | 96 | 978 | 16 | 28 | 176 | 10 | 93.5 | 15500 | n.d. |
| 06_08302024_WCapitolSt_Site2 | 5 | 89.7 | 474 | 20.3 | 72 | 599 | 22 | 663 | 22000 | n.d. |
| 07_08122024_GlenErinSt | 6 | 67.3 | 423 | 14.7 | 7.67 | 79.7 | 7.67 | 30.7 | 15300 | n.d. |
| 08_08122024_MeadowLn | 6 | 80 | 764 | 24.3 | 41.7 | 250 | 23.3 | 59.7 | 18300 | n.d. |
| 09_11142024_MetroCenter | 5 | 90 | 908 | 22 | 17.5 | 105 | 11.5 | 38 | 21100 | n.d. |
| 10_11142024_TeresaDr | 6 | 104 | 378 | 22.5 | 34.5 | 184 | 13.5 | 51.5 | 23100 | n.d. |
| 11_11142024_RevereSt | 6 | 106 | 469 | 38 | 205 | 253 | 14.5 | 81 | 23200 | n.d. |
| 12_11142024_CooperRd | 6 | 104 | 696 | 27 | 130 | 376 | 25 | 94 | 24100 | n.d. |
| 13_11142024_ForestHillRd | 6 | 101 | 287 | 19 | 28 | 142 | 10.3 | 53.3 | 19800 | n.d. |
| 14_11142024_MccluerRd | 6 | 95.5 | 602 | 26.5 | 17 | 86.5 | 13.5 | 50 | 22500 | n.d. |
| 15_11142024_RobinsonRd | 5 | 63.7 | 396 | 16.3 | 13.3 | 60 | 15 | 13 | 15000 | n.d. |
| 16_11142024_BarnettDr | 5 | 101 | 409 | 25 | 14.3 | 100 | 11.7 | 32 | 25000 | n.d. |
| 17_11142024_NPrentissSt | 5 | 101 | 502 | 22.3 | 21.3 | 400 | 17.7 | 351 | 24100 | n.d. |
| 18_11142024_LongSt | 5 | 113 | 656 | 22.3 | 29.7 | 201 | 12.3 | 102 | 23600 | n.d. |
| 19_11142024_BonAirSt | 5 | 100 | 396 | 22 | 165 | 222 | 17.7 | 168 | 22700 | n.d. |
| 20_11142024_CollierAve | 3 | 95 | 1080 | 27 | 76 | 323 | 17.5 | 205 | 21100 | n.d. |
| 22_11142024_MartinLutherKingJrDr | 5 | 64 | 356 | 14.5 | 19 | 182 | 10 | 202 | 10700 | n.d. |
| 23_11142024_JohnRLynchSt | 7 | 93.5 | 636 | 22.3 | 70.7 | 495 | 23.7 | 616 | 21100 | n.d. |
| 24_11142024_CliftonSt | 7 | 113 | 1230 | 24 | 75 | 962 | 44.5 | 2770 | 24600 | n.d. |
| 25_11142024_HookerSt | 7 | 75 | 921 | 21.7 | 1220 | 591 | 10.3 | 538 | 16000 | n.d. |
| 26_11142024_WCountyLineRd_Site1 | 2 | 91 | 847 | 19 | 11.3 | 85.3 | 9.67 | 33.3 | 18200 | n.d. |
| 27_11142024_BrownSt | 2 | 91.3 | 470 | 25 | 17 | 106 | 11 | 26 | 20500 | n.d. |
| 28_11142024_GilbertAve | 2 | 104 | 616 | 27.5 | 17.5 | 124 | 15 | 26 | 25800 | n.d. |
| 29_11142024_Highway49_Site1 | 2 | 100 | 611 | 34.5 | 127 | 372 | 14 | 62 | 25500 | n.d. |
| 32_11142024_WinterSt | 5 | 122 | 572 | 24 | 75 | 430 | 12.5 | 138 | 17600 | n.d. |
| 33_11142024_ClearySt | 5 | 106 | 438 | 30 | 80 | 504 | 15 | 220 | 24400 | n.d. |
| 34_11142024_MedgarEversBlvd_Site1 | 3 | 109 | 852 | 31 | 23.5 | 116 | 10.5 | 32 | 32900 | n.d. |
| 35_11142024_MedgarEversBlvd_Site2 | 3 | 118 | 634 | 27.5 | 46 | 383 | 22.5 | 128 | 20600 | n.d. |
| 36_02212025_JohnsonSt | 3 | 81 | 390 | 19 | 15 | 196 | 12 | 57 | 17300 | n.d. |
| 39_02212025_WMcDowellRd_Site1 | 7 | 111 | 969 | 27.7 | 217 | 183 | 12 | 53 | 21600 | n.d. |
| 42_02212025_FlorenceAve | 5 | 57.5 | 360 | 19.5 | 26.5 | 222 | 8 | 91 | 16800 | n.d. |
| 49_02212025_SunrayDr | 4 | 91 | 632 | 28 | 55 | 429 | 17.3 | 490 | 20700 | n.d. |
| 50_02212025_PercyVSimpsonDr | 3 | 128 | 572 | 40.7 | 88.3 | 351 | 13.7 | 110 | 23700 | n.d. |
| 51_02212025_KingswoodAve | 7 | 83 | 506 | 23.7 | 98.3 | 513 | 10.3 | 66.7 | 20600 | n.d. |
| 52_02212025_LaSalleSt | 7 | 103 | 1050 | 28.3 | 96.3 | 252 | 11.3 | 84.7 | 20200 | n.d. |
| 53_02212025_RoseSt | 5 | 87.5 | 531 | 25 | 52 | 394 | 10 | 55.5 | 21900 | n.d. |
| 55_06252025_WCountyLineRd_Site2 | 2 | 173 | 651 | 51.3 | 97.3 | 320 | 12.3 | 158 | 26400 | n.d. |
| 56_06252025_BrownSt_Site 2 | 2 | 49 | 1130 | 17.5 | 13 | 154 | 6.5 | 25 | 13000 | n.d. |
| 57_06302025_StateSt_Site1 | 3 | 72.5 | 486 | 19.5 | 20 | 101 | 12.5 | 58 | 17300 | n.d. |
| 61_06242025_ShirleyAve_Site1 | 5 | 148 | 514 | 29 | 268 | 711 | 10.3 | 67.3 | 17500 | 18 |
| 62_06252025_HildaDr_Site1 | 2 | 105 | 689 | 22 | 47.7 | 155 | 11.3 | 61 | 20900 | n.d. |

*Determined by portable XRF. n.d., not detected (below the instrument reporting threshold). Iron is reported from the 90 s acquisition.*

**Table S4d** Element concentrations at 15 cm (mg/kg) for all 45 sites

| **Site ID** | **Ward** | **Cr** | **Mn** | **Ni** | **Cu** | **Zn** | **As** | **Pb** | **Fe** | **Cd** |
| --- | --- | --- | --- | --- | --- | --- | --- | --- | --- | --- |
| 01_06042024_MinervaSt | 5 | 96 | 682 | 24 | 174 | 731 | 28 | 544 | 28900 | n.d. |
| 02_06122024_HartfieldSt | 7 | 105 | 1470 | 28.3 | 49 | 317 | 14.7 | 93 | 20500 | n.d. |
| 03_06242024_ClaiborneAve | 2 | 75 | 657 | 20 | 316 | 835 | 25.3 | 396 | 17000 | n.d. |
| 04_07162024_WCapitolSt_Site1 | 5 | 83 | 488 | 22 | 37.5 | 328 | 15.5 | 427 | 19200 | n.d. |
| 05_07302024_ByrdDr | 4 | 69 | 953 | 16.7 | 16.7 | 150 | 9.67 | 119 | 15800 | n.d. |
| 06_08302024_WCapitolSt_Site2 | 5 | 100 | 506 | 26.3 | 106 | 541 | 18.3 | 587 | 22500 | n.d. |
| 07_08122024_GlenErinSt | 6 | 82.7 | 334 | 18 | 7.67 | 66 | 9.67 | 27.7 | 16200 | n.d. |
| 08_08122024_MeadowLn | 6 | 94.3 | 654 | 21.7 | 32 | 275 | 19 | 68.7 | 19400 | n.d. |
| 09_11142024_MetroCenter | 5 | 112 | 623 | 22 | 11.5 | 106 | 12 | 47 | 22500 | n.d. |
| 10_11142024_TeresaDr | 6 | 103 | 289 | 22 | 20 | 253 | 14 | 51.5 | 23500 | n.d. |
| 11_11142024_RevereSt | 6 | 113 | 410 | 47.7 | 194 | 151 | 13 | 78.7 | 24900 | n.d. |
| 12_11142024_CooperRd | 6 | 128 | 603 | 28 | 78.7 | 314 | 23 | 103 | 23300 | n.d. |
| 13_11142024_ForestHillRd | 6 | 116 | 175 | 15.7 | 15 | 103 | 8.33 | 63.3 | 19800 | n.d. |
| 14_11142024_MccluerRd | 6 | 109 | 520 | 27 | 16.7 | 84.3 | 15 | 38.3 | 26200 | n.d. |
| 15_11142024_RobinsonRd | 5 | 77 | 433 | 21.3 | 11.7 | 62 | 12.7 | 22 | 17900 | n.d. |
| 16_11142024_BarnettDr | 5 | 108 | 522 | 26.7 | 16.7 | 89.7 | 14.7 | 37.7 | 27000 | n.d. |
| 17_11142024_NPrentissSt | 5 | 120 | 441 | 23.3 | 21 | 386 | 26 | 330 | 23700 | n.d. |
| 18_11142024_LongSt | 5 | 117 | 590 | 23.7 | 24.3 | 183 | 15 | 139 | 25300 | n.d. |
| 19_11142024_BonAirSt | 5 | 105 | 331 | 22.7 | 126 | 157 | 12 | 66.7 | 22700 | 19 |
| 20_11142024_CollierAve | 3 | 134 | 1320 | 27.7 | 158 | 310 | 26.7 | 261 | 21100 | n.d. |
| 22_11142024_MartinLutherKingJrDr | 5 | 53 | 750 | 14.3 | 11.3 | 147 | 10.3 | 162 | 11100 | n.d. |
| 23_11142024_JohnRLynchSt | 7 | 79.5 | 624 | 17.5 | 72 | 504 | 23.5 | 669 | 21300 | n.d. |
| 24_11142024_CliftonSt | 7 | 125 | 1290 | 25.3 | 82.3 | 1070 | 53 | 2910 | 25600 | 27 |
| 25_11142024_HookerSt | 7 | 88.3 | 999 | 24 | 406 | 352 | 13.7 | 285 | 18200 | n.d. |
| 26_11142024_WCountyLineRd_Site1 | 2 | 104 | 870 | 25.7 | 13 | 87 | 11.7 | 35.7 | 18700 | n.d. |
| 27_11142024_BrownSt | 2 | 99.7 | 356 | 23 | 16 | 81.7 | 12.3 | 17.7 | 22400 | n.d. |
| 28_11142024_GilbertAve | 2 | 128 | 615 | 24 | 14.7 | 101 | 13.7 | 27.3 | 28100 | n.d. |
| 29_11142024_Highway49_Site1 | 2 | 112 | 580 | 33 | 134 | 344 | 14.5 | 64.5 | 28200 | n.d. |
| 32_11142024_WinterSt | 5 | 182 | 501 | 23.5 | 75.5 | 408 | 12.5 | 116 | 17500 | n.d. |
| 33_11142024_ClearySt | 5 | 109 | 414 | 30 | 161 | 546 | 17.8 | 227 | 24700 | n.d. |
| 34_11142024_MedgarEversBlvd_Site1 | 3 | 119 | 872 | 30.5 | 22.5 | 116 | 10.5 | 29 | 33200 | n.d. |
| 35_11142024_MedgarEversBlvd_Site2 | 3 | 99.7 | 685 | 25 | 44 | 371 | 21.3 | 125 | 20100 | n.d. |
| 36_02212025_JohnsonSt | 3 | 78.5 | 538 | 22.5 | 19 | 186 | 11 | 65 | 18200 | n.d. |
| 39_02212025_WMcDowellRd_Site1 | 7 | 126 | 850 | 26.8 | 168 | 190 | 11.5 | 57.7 | 21200 | n.d. |
| 42_02212025_FlorenceAve | 5 | 65.5 | 402 | 19.5 | 30 | 203 | 10 | 114 | 17000 | n.d. |
| 49_02212025_SunrayDr | 4 | 81 | 642 | 29 | 108 | 756 | 13.7 | 721 | 20800 | n.d. |
| 50_02212025_PercyVSimpsonDr | 3 | 127 | 496 | 39.7 | 68.7 | 344 | 14.7 | 128 | 28500 | n.d. |
| 51_02212025_KingswoodAve | 7 | 103 | 734 | 27 | 144 | 708 | 13.3 | 97.3 | 21400 | n.d. |
| 52_02212025_LaSalleSt | 7 | 125 | 1060 | 27.7 | 124 | 260 | 10 | 78 | 18600 | n.d. |
| 53_02212025_RoseSt | 5 | 93.5 | 476 | 21.5 | 41.5 | 365 | 10 | 51.5 | 20700 | n.d. |
| 55_06252025_WCountyLineRd_Site2 | 2 | 155 | 632 | 32.7 | 46.7 | 155 | 15.3 | 52.7 | 23600 | n.d. |
| 56_06252025_BrownSt_Site 2 | 2 | 63.5 | 3680 | 55 | 68 | 316 | 8 | 25 | 14400 | 24 |
| 57_06302025_StateSt_Site1 | 3 | 84 | 436 | 16 | 20 | 91 | 11.5 | 54.5 | 16800 | n.d. |
| 61_06242025_ShirleyAve_Site1 | 5 | 111 | 505 | 27.7 | 40.7 | 131 | 13 | 53 | 17600 | n.d. |
| 62_06252025_HildaDr_Site1 | 2 | 90.3 | 564 | 28.7 | 89.7 | 450 | 12 | 210 | 22800 | n.d. |

*Determined by portable XRF. n.d., not detected.*

**Table S4e** Contamination factors and degree of contamination for all 45 sites

| **Site ID** | **Ward** | **Cf Cr** | **Cf Mn** | **Cf Ni** | **Cf Cu** | **Cf Zn** | **Cf As** | **Cf Pb** | **Cdeg** | **Class** |
| --- | --- | --- | --- | --- | --- | --- | --- | --- | --- | --- |
| 01_06042024_MinervaSt | 5 | 2.42 | 1.19 | 0.741 | 13.4 | 18.1 | 3.99 | 20.7 | 60.5 | High |
| 02_06122024_HartfieldSt | 7 | 2.79 | 2.9 | 0.871 | 3.36 | 8.2 | 1.86 | 3.53 | 23.5 | Considerable |
| 03_06242024_ClaiborneAve | 2 | 2.44 | 1.66 | 0.697 | 25.4 | 31.7 | 4.08 | 20.6 | 86.6 | High |
| 04_07162024_WCapitolSt_Site1 | 5 | 2.29 | 0.919 | 0.727 | 3.27 | 10.1 | 2.5 | 19.3 | 39.1 | High |
| 05_07302024_ByrdDr | 4 | 2.2 | 1.9 | 0.534 | 1.56 | 4.19 | 1.43 | 4.04 | 15.9 | Moderate |
| 06_08302024_WCapitolSt_Site2 | 5 | 2.53 | 0.963 | 0.763 | 6.22 | 14.6 | 2.92 | 23.8 | 51.8 | High |
| 07_08122024_GlenErinSt | 6 | 2 | 0.744 | 0.534 | 0.536 | 1.87 | 1.26 | 1.11 | 8.05 | Moderate |
| 08_08122024_MeadowLn | 6 | 2.32 | 1.39 | 0.752 | 2.58 | 6.75 | 3.07 | 2.44 | 19.3 | Considerable |
| 09_11142024_MetroCenter | 5 | 2.69 | 1.5 | 0.719 | 1.01 | 2.71 | 1.7 | 1.62 | 12 | Moderate |
| 10_11142024_TeresaDr | 6 | 2.76 | 0.656 | 0.727 | 1.91 | 5.62 | 1.99 | 1.96 | 15.6 | Moderate |
| 11_11142024_RevereSt | 6 | 2.92 | 0.863 | 1.4 | 14 | 5.19 | 1.99 | 3.04 | 29.3 | Considerable |
| 12_11142024_CooperRd | 6 | 3.1 | 1.28 | 0.899 | 7.31 | 8.87 | 3.48 | 3.75 | 28.7 | Considerable |
| 13_11142024_ForestHillRd | 6 | 2.89 | 0.454 | 0.566 | 1.5 | 3.14 | 1.35 | 2.22 | 12.1 | Moderate |
| 14_11142024_MccluerRd | 6 | 2.72 | 1.1 | 0.874 | 1.18 | 2.2 | 2.07 | 1.68 | 11.8 | Moderate |
| 15_11142024_RobinsonRd | 5 | 1.88 | 0.814 | 0.615 | 0.874 | 1.57 | 2 | 0.665 | 8.42 | Moderate |
| 16_11142024_BarnettDr | 5 | 2.8 | 0.914 | 0.844 | 1.08 | 2.44 | 1.91 | 1.32 | 11.3 | Moderate |
| 17_11142024_NPrentissSt | 5 | 2.94 | 0.926 | 0.746 | 1.48 | 10.1 | 3.16 | 12.9 | 32.3 | High |
| 18_11142024_LongSt | 5 | 3.07 | 1.22 | 0.752 | 1.89 | 4.93 | 1.98 | 4.58 | 18.4 | Considerable |
| 19_11142024_BonAirSt | 5 | 2.73 | 0.713 | 0.73 | 10.2 | 4.88 | 2.15 | 4.45 | 25.8 | Considerable |
| 20_11142024_CollierAve | 3 | 3.06 | 2.36 | 0.893 | 8.18 | 8.14 | 3.2 | 8.87 | 34.7 | High |
| 22_11142024_MartinLutherKingJrDr | 5 | 1.56 | 1.09 | 0.471 | 1.06 | 4.22 | 1.47 | 6.94 | 16.8 | Considerable |
| 23_11142024_JohnRLynchSt | 7 | 2.31 | 1.24 | 0.651 | 4.99 | 12.8 | 3.42 | 24.4 | 49.9 | High |
| 24_11142024_CliftonSt | 7 | 3.16 | 2.48 | 0.806 | 5.5 | 26.1 | 7.07 | 108 | 153 | High |
| 25_11142024_HookerSt | 7 | 2.18 | 1.89 | 0.746 | 56.9 | 12.1 | 1.74 | 15.6 | 91.3 | High |
| 26_11142024_WCountyLineRd_Site1 | 2 | 2.6 | 1.69 | 0.73 | 0.851 | 2.22 | 1.55 | 1.31 | 10.9 | Moderate |
| 27_11142024_BrownSt | 2 | 2.55 | 0.811 | 0.784 | 1.15 | 2.41 | 1.69 | 0.83 | 10.2 | Moderate |
| 28_11142024_GilbertAve | 2 | 3.1 | 1.21 | 0.842 | 1.12 | 2.88 | 2.08 | 1.01 | 12.3 | Moderate |
| 29_11142024_Highway49_Site1 | 2 | 2.82 | 1.17 | 1.1 | 9.13 | 9.2 | 2.07 | 2.4 | 27.9 | Considerable |
| 32_11142024_WinterSt | 5 | 4.05 | 1.05 | 0.776 | 5.26 | 10.8 | 1.81 | 4.83 | 28.5 | Considerable |
| 33_11142024_ClearySt | 5 | 2.86 | 0.837 | 0.98 | 8.43 | 13.5 | 2.37 | 8.51 | 37.5 | High |
| 34_11142024_MedgarEversBlvd_Site1 | 3 | 3.04 | 1.69 | 1 | 1.61 | 2.98 | 1.52 | 1.16 | 13 | Moderate |
| 35_11142024_MedgarEversBlvd_Site2 | 3 | 2.9 | 1.3 | 0.858 | 3.15 | 9.7 | 3.18 | 4.82 | 25.9 | Considerable |
| 36_02212025_JohnsonSt | 3 | 2.13 | 0.912 | 0.678 | 1.19 | 4.92 | 1.67 | 2.32 | 13.8 | Moderate |
| 39_02212025_WMcDowellRd_Site1 | 7 | 3.16 | 1.79 | 0.889 | 13.5 | 4.79 | 1.7 | 2.1 | 27.9 | Considerable |
| 42_02212025_FlorenceAve | 5 | 1.64 | 0.748 | 0.637 | 1.98 | 5.46 | 1.3 | 3.9 | 15.7 | Moderate |
| 49_02212025_SunrayDr | 4 | 2.29 | 1.25 | 0.931 | 5.69 | 15.2 | 2.25 | 23 | 50.7 | High |
| 50_02212025_PercyVSimpsonDr | 3 | 3.4 | 1.05 | 1.31 | 5.49 | 8.93 | 2.05 | 4.53 | 26.8 | Considerable |
| 51_02212025_KingswoodAve | 7 | 2.48 | 1.22 | 0.828 | 8.46 | 15.7 | 1.71 | 3.12 | 33.5 | High |
| 52_02212025_LaSalleSt | 7 | 3.04 | 2.07 | 0.915 | 7.69 | 6.58 | 1.55 | 3.09 | 24.9 | Considerable |
| 53_02212025_RoseSt | 5 | 2.41 | 0.989 | 0.76 | 3.27 | 9.76 | 1.45 | 2.03 | 20.7 | Considerable |
| 55_06252025_WCountyLineRd_Site2 | 2 | 4.36 | 1.26 | 1.37 | 5.03 | 6.11 | 2 | 4.01 | 24.2 | Considerable |
| 56_06252025_BrownSt_Site 2 | 2 | 1.5 | 4.73 | 1.18 | 2.83 | 6.03 | 1.05 | 0.951 | 18.3 | Considerable |
| 57_06302025_StateSt_Site1 | 3 | 2.09 | 0.905 | 0.58 | 1.4 | 2.47 | 1.74 | 2.14 | 11.3 | Moderate |
| 61_06242025_ShirleyAve_Site1 | 5 | 3.44 | 1 | 0.926 | 10.8 | 10.8 | 1.69 | 2.29 | 31 | Considerable |
| 62_06252025_HildaDr_Site1 | 2 | 2.6 | 1.23 | 0.828 | 4.8 | 7.77 | 1.69 | 5.15 | 24.1 | Considerable |

*Cf is the ratio of the depth-averaged concentration to the reference background value (main text Table 1). Cdeg is the sum of the seven contamination factors and is classified after Hakanson (1980) as low (below 8), moderate (8 to 16), considerable (16 to 32) or high (32 or above). No site fell in the low class.*

**Table S5** Portable XRF measurement reproducibility, assessed by comparison of 60 s and 90 s acquisitions

| **Element** | **Depth (cm)** | **n** | **Pearson r** | **Mean relative difference (%)** | **Maximum relative difference (%)** | **Samples differing by more than 25 %** |
| --- | --- | --- | --- | --- | --- | --- |
| Cr | 0 to 2.5 | 45 | 0.906 | 8.49 | 31.6 | 3 |
| Cr | 15 | 45 | 0.902 | 8.91 | 37.4 | 2 |
| Mn | 0 to 2.5 | 45 | 0.993 | 4.15 | 12.3 | 0 |
| Mn | 15 | 45 | 0.963 | 5.07 | 56.3 | 2 |
| Ni | 0 to 2.5 | 45 | 0.904 | 10.75 | 34.5 | 4 |
| Ni | 15 | 45 | 0.628 | 15.08 | 111.5 | 4 |
| Cu | 0 to 2.5 | 45 | 0.998 | 13.66 | 69.6 | 10 |
| Cu | 15 | 45 | 0.980 | 15.09 | 115.9 | 9 |
| Zn | 0 to 2.5 | 45 | 0.999 | 3.50 | 14.2 | 0 |
| Zn | 15 | 45 | 0.994 | 5.96 | 87.4 | 2 |
| As | 0 to 2.5 | 45 | 0.915 | 21.67 | 87.5 | 14 |
| As | 15 | 45 | 0.924 | 17.48 | 66.7 | 11 |
| Pb | 0 to 2.5 | 45 | 0.999 | 7.83 | 37.0 | 2 |
| Pb | 15 | 45 | 1.000 | 7.26 | 39.6 | 2 |
| Fe | 0 to 2.5 | 45 | 0.863 | 45.23 | 1959.3 | 1 |
| Fe | 15 | 45 | 0.990 | 2.03 | 19.8 | 0 |

*Reproducibility is poorest for arsenic, copper and nickel, the elements present at the lowest concentrations, which is the basis for interpreting the portable XRF data as screening-level. The single large iron discrepancy at the surface arises from one failed 60 s acquisition at a site reading 886 mg/kg against 18,245 mg/kg at 90 s; iron is therefore reported from the 90 s acquisition throughout. No certified reference material was analysed by portable XRF.*

**Quality assurance for ICP-OES analysis**

Analyses were performed by Waters Agricultural Laboratories, Inc. (Vicksburg, Mississippi, USA) on a PerkinElmer Optima 8000. The certified reference material NIST SRM 2711a was digested and analysed with each batch, with recoveries ranging from 94.8 to 103.2 % against a laboratory acceptance window of 90 to 110 %. Calibration used six points plus a blank prepared from multi-element standards traceable to the National Institute of Standards and Technology and manufactured under ISO/IEC 17025 and ISO 17034 quality management systems, accepted at a coefficient of determination of 0.999 or better. Continuing calibration verification standards were analysed approximately every 10 to 20 samples and accepted within 90 to 110 % of the expected value. Method blanks, laboratory duplicates and matrix spikes were also analysed. Element-specific reporting limits were not provided in the laboratory report.

**Table S6** Pearson correlations between element concentrations and total iron

| **Variable** | **r with Fe (0 to 2.5 cm)** | **p** | **r with Fe (15 cm)** | **p** |
| --- | --- | --- | --- | --- |
| Cr, 0 to 2.5 cm | 0.520 | 2.5 × 10⁻⁴ | 0.426 | 3.6 × 10⁻³ |
| Cr, 15 cm | 0.547 | 1.0 × 10⁻⁴ | 0.495 | 5.5 × 10⁻⁴ |
| Mn, 0 to 2.5 cm | 0.000 | 0.999 | −0.037 | 0.811 |
| Mn, 15 cm | −0.237 | 0.117 | −0.225 | 0.137 |
| Ni, 0 to 2.5 cm | 0.585 | 2.4 × 10⁻⁵ | 0.549 | 9.3 × 10⁻⁵ |
| Ni, 15 cm | 0.226 | 0.136 | 0.301 | 0.045 |
| Cu, 0 to 2.5 cm | −0.100 | 0.511 | −0.084 | 0.583 |
| Cu, 15 cm | 0.041 | 0.788 | 0.017 | 0.910 |
| Zn, 0 to 2.5 cm | 0.077 | 0.615 | −0.022 | 0.888 |
| Zn, 15 cm | 0.186 | 0.221 | 0.118 | 0.441 |
| As, 0 to 2.5 cm | 0.304 | 0.043 | 0.239 | 0.114 |
| As, 15 cm | 0.338 | 0.023 | 0.288 | 0.055 |
| Pb, 0 to 2.5 cm | 0.138 | 0.366 | 0.105 | 0.494 |
| Pb, 15 cm | 0.160 | 0.293 | 0.138 | 0.364 |

*Iron is reported from the 90 s acquisition. Chromium and nickel co-vary significantly with iron and arsenic weakly so, whereas copper, zinc, lead and manganese show no association at either depth. Correlations between the measured elements and survey-derived organic matter were not significant at either depth; because those data are mapped attributes at map-unit resolution rather than sample determinations, that result is inconclusive rather than evidence against an organic matter association.*

**Table S7** Soil series, texture and pH class distribution across the 45 sites

| **Category** | **Class** | **Number of sites** |
| --- | --- | --- |
| Soil series (survey map unit) | Loring-Urban | 18 |
|  | Byram-Urban | 6 |
|  | Riedtown | 6 |
|  | Siwell-Urban | 6 |
|  | Byram | 2 |
|  | Calloway-Urban | 2 |
|  | Adler | 1 |
|  | Loring-Memphis | 1 |
|  | Oaklimeter | 1 |
|  | Siwell | 1 |
|  | Urban Land | 1 |
| Texture, 0 to 2.5 cm | Silt loam | 43 |
|  | Clay loam | 1 |
|  | Silt | 1 |
| Texture, 15 cm | Silt loam | 31 |
|  | Silty clay loam | 13 |
|  | Clay loam | 1 |
| Soil pH class | Acidic | 28 |
|  | Neutral | 12 |
|  | Alkaline | 5 |

*Soil series and texture are survey-derived attributes. Soil pH was measured in the field with a probe and ranged from 3.0 to 8.7 across the 45 sites.*
